# A proof-of-concept study on bioorthogonal-based pretargeting and signal amplify radiotheranostic strategy

**DOI:** 10.1186/s12951-024-02312-y

**Published:** 2024-03-10

**Authors:** Hongzhang Yang, Xinying Zeng, Jia Liu, Jingchao Li, Yun Li, Qinglin Zhang, Linlin Shu, Huanhuan Liu, Xueqi Wang, Yuanyuan Liang, Ji Hu, Lumei Huang, Zhide Guo, Xianzhong Zhang

**Affiliations:** 1 State Key Laboratory of Vaccines for Infectious Diseases, Center for Molecular Imaging and Translational Medicine, Xiang An Biomedicine Laboratory, School of Public Health, Xiamen University, Xiamen, 361102 China; 2 Theranostics and Translational Research Center, Institute of Clinical Medicine & Department of Nuclear Medicine, Peking Union Medical College Hospital, Chinese Academy of Medical Sciences & Peking Union Medical College, No.1 Shuaifuyuan, Dongcheng District, Beijing, 100730 China; 3 PET Center, Department of Nuclear Medicine, School of Medicine, The First Affiliated Hospital, Zhejiang University, 79 Qingchun Road, Hangzhou, 310003 China; 4 HTA Co., Ltd., No. 1 Sanqiang Road, Fangshan District, Beijing, 102413 China

**Keywords:** Radiotheranostics, Click-mediated bioorthogonal chemistry, Tumor signal amplification, Pretargeting, PSMA

## Abstract

**Background:**

Radiotheranostics differs from the vast majority of other cancer therapies in its capacity for simultaneous imaging and therapy, and it is becoming more widely implemented. A balance between diagnostic and treatment requirements is essential for achieving effective radiotheranostics. Herein, we propose a proof-of-concept strategy aiming to address the profound differences in the specific requirements of the diagnosis and treatment of radiotheranostics.

**Results:**

To validate the concept, we designed an *s*-tetrazine (Tz) conjugated prostate-specific membrane antigen (PSMA) ligand (DOTA-PSMA-Tz) for ^68^Ga or ^177^Lu radiolabeling and tumor radiotheranostics, a *trans*-cyclooctene (TCO) modified Pd@Au nanoplates (Pd@Au-PEG-TCO) for signal amplification, respectively. We then demonstrated this radiotheranostic strategy in the tumor-bearing mice with the following three-step procedures: (1) i.v. injection of the [^68^Ga]Ga-PSMA-Tz for diagnosis; (2) i.v. injection of the signal amplification module Pd@Au-PEG-TCO; (3) i.v. injection of the [^177^Lu]Lu-PSMA-Tz for therapy. Firstly, this strategy was demonstrated in 22Rv1 tumor-bearing mice via positron emission tomography (PET) imaging with [^68^Ga]Ga-PSMA-Tz. We observed significantly higher tumor uptake (11.5 ± 0.8%ID/g) with the injection of Pd@Au-PEG-TCO than with the injection [^68^Ga]Ga-PSMA-Tz alone (5.5 ± 0.9%ID/g). Furthermore, we validated this strategy through biodistribution studies of [^177^Lu]Lu-PSMA-Tz, with the injection of the signal amplification module, approximately five-fold higher tumor uptake of [^177^Lu]Lu-PSMA-Tz (24.33 ± 2.53% ID/g) was obtained when compared to [^177^Lu]Lu-PSMA-Tz alone (5.19 ± 0.26%ID/g) at 48 h post-injection.

**Conclusion:**

In summary, the proposed strategy has the potential to expand the toolbox of pretargeted radiotherapy in the field of theranostics.

**Graphical Abstract:**

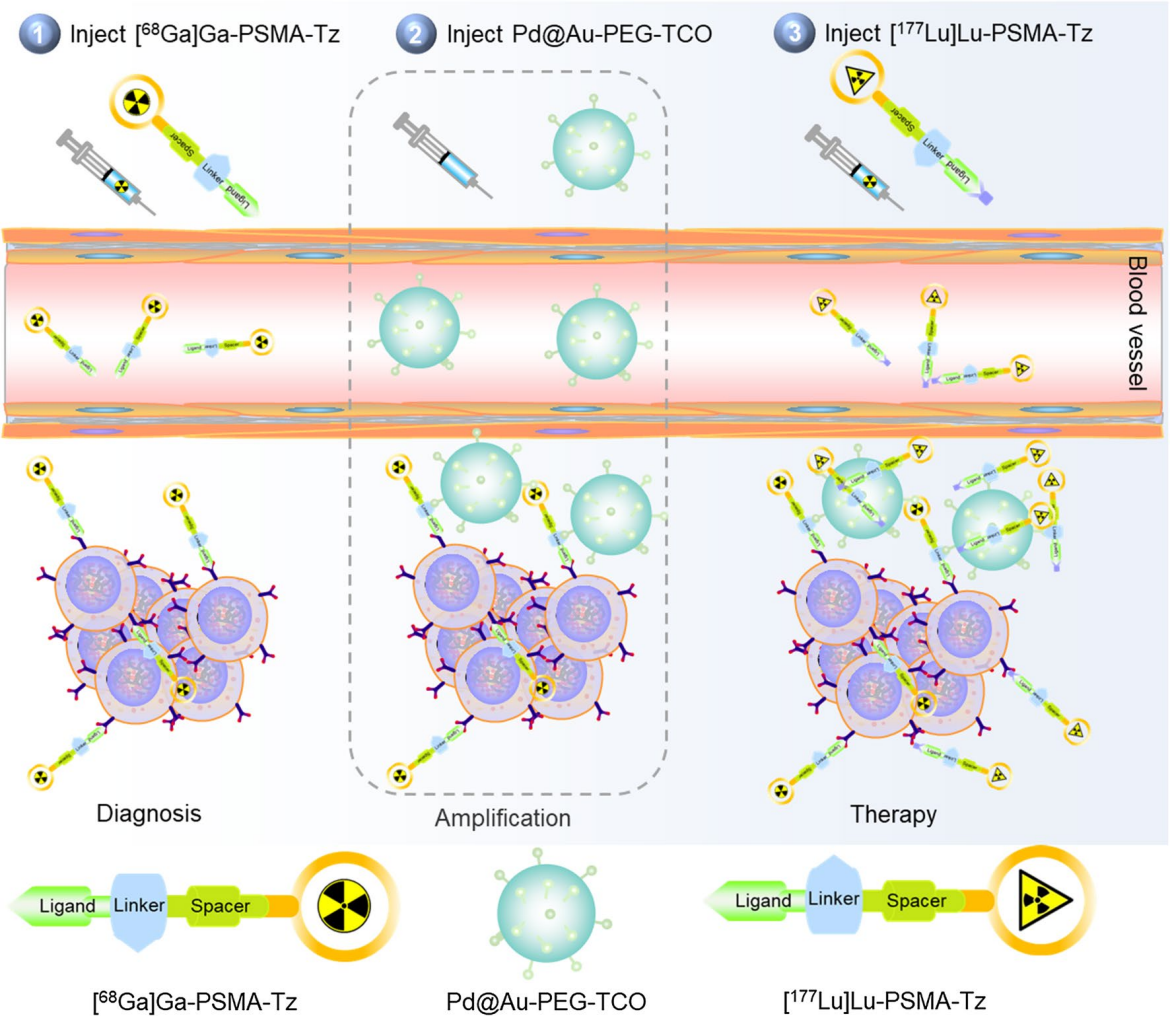

**Supplementary Information:**

The online version contains supplementary material available at 10.1186/s12951-024-02312-y.

## Introduction

Theranostics, which involves collaborative designs of diagnostic and therapeutic tracers, describes a close interplay between diagnostics and therapy, to provide personalized precision medicine for patients [1–3]. Targeted radionuclide therapy (TRT) has emerged as a promising strategy for theranostics via versatile radionuclides such as ^64^Cu, ^177^Lu, and ^131^I, etc. [4–7] The primary goal of tumor diagnosis is to achieve high-contrast images with a high target-to-non-target ratio, whilst ensuring efficient in vivo clearance of radiopharmaceuticals [8]. However, there exists no stringent criterion for the absolute tumor uptake of radiopharmaceuticals. On the other hand, high uptake and prolonged retention of radiopharmaceuticals at the tumor site are necessary for tumor therapy to ensure treatment efficacy [9–11]. Although traditional dual-purpose radiotracers successfully integrate diagnosis and therapy within one tracer, their separate efficacy for each function may not be optimal. For instance, ^177^Lu is more suitable for therapy than imaging, and ^64^Cu is preferred for imaging due to its limited beta-therapeutic efficacy [12, 13]. Given the aforementioned challenges, the feasibility of integrating the diagnostic and therapeutic modules within a single platform warrants further research, and considerable effort needs to be dedicated to developing strategies to meet completely different requirements of diagnostics and therapies in TRT.

Pre-targeted radiotherapy (PRT) represents an alternative elegant strategy for ameliorating TRT by integrating the high specificity and affinity of antibodies and the rapid pharmacokinetics of small molecules [14–18]. In vivo pre-targeting achieves this by decoupling the targeting vector and radioactivity, administering the two components separately, and allowing them to be coupled within the body. Due to the high reaction rate (*k*_2_ up to 10^6^ M^−1^ s^−1^), non-metallic nature, and physiological pH, the bioorthogonal ligation of *s*-tetrazine (Tz) and *trans*-cyclooctene (TCO) derivatives have been broadly used in PRT [19, 20]. The implementation of in vivo pre-targeted technology is an efficient approach to avoid the prolonged circulation of radionuclides with the carrier in the body, thereby expanding the spectrum of radionuclide applications. Furthermore, this strategy potentiates the differentiation of diagnostic and therapeutic radiopharmaceuticals, serving not only to aid patient selection for pre-targeted radioimmunotherapy (PRIT) but also to optimize the dose and therapeutic schedule of PRIT regimens [21, 22]. Additionally, this separation allows the utilization of more suitable radionuclides during different stages, which is of paramount importance. Notwithstanding the efficacy of the elegant strategy in resolving the challenges associated with radionuclides’ protracted system circulation and unsuitable radionuclides, there exists a dearth of comprehensive investigations pertaining to the variance in tumor uptake levels necessary for therapeutic and diagnostic purposes. [23]

As mentioned above, it is important to achieve amplified signals at the tumor site and low signals in healthy organs, which has attracted increasing attention from researchers worldwide. Most notably Brian M. Zeglis, et al. have exploited two recent developments in pre-targeted imaging—dendritic scaffolds and masking agents—to improve the dosimetric profile of a PRIT system [24]. Pre-targeting via* trans*-cyclooctene-decorated dendritic scaffold-modified huA33 immunoconjugate produced significantly high tumor uptake, and a dextran-based masking agent modified with tetrazine dramatically decreased the absorbed dose in the blood. Unfortunately, the addition of the masking agent resulted in attenuated tumor uptake and increased lung uptake. Therefore, effective strategies for signal amplification in PRT require much more intensive and systematic studies.

In this study, we proposed a proof-of-concept strategy that combines tumor pre-targeting and signal amplification based on click-mediated bioorthogonal chemistry, to amplify tumor signals for TRT. The three-step procedure includes (1) nuclear medicine imaging with a tumor-targeting radiopharmaceutical that contains the Tz group for tumor diagnostic and functional pre-targeting; (2) injection of TCO-bearing signal amplification module, which accumulated in the tumor via enhanced permeability and retention (EPR) effect and/or click reaction with the pretargeted Tz group; (3) injection of therapeutic radiopharmaceuticals for tumor TRT and enhanced tumor uptake is expected to be obtained through Tz-TCO click-reaction with accumulated TCO group in the tumor site (Graphical abstract). Specifically, a Tz-conjugated prostate-specific membrane antigen (PSMA) targeting ligand, denoted as DOTA-PSMA-Tz, was developed and radiolabeled with radionuclide ^68^Ga ([^68^Ga]Ga-PSMA-Tz) or ^177^Lu ([^177^Lu]Lu-PSMA-Tz) as example and evaluated for its tumor-targeting effectiveness both in vitro and in vivo. [25, 26] Concurrently, a signal amplification module based on TCO-bearing PEGylated 2D Pd@Au nanoplates, which have distinct metabolic profiles from DOTA-PSMA-Tz, significantly accumulates in the tumor owing to the EPR effect [27]. Taking into account the distinctive metabolism models of DOTA-PSMA-Tz and Pd@Au-PEG-TCO, signal amplification exclusively occurred within the tumor. In addition, the presence of Pd@Au-PEG-TCO provides an abundance of active binding sites for the dual-targeting DOTA-PSMA-Tz during the third step, whereby PSMA and Pd@Au-PEG-TCO were effectively targeted to enhance its tumor uptake and improve the target-to-non-target (T/NT) ratios. Exactly, the signal amplification system provides an intelligent paradigm to realize safe and reliable pre-targeted radiotherapy for theranostics.

## Materials and methods

### Materials

All the reagents we used in the synthesis and biology experiment were purchased from Energy Chemical Co., Ltd. (China) or J&K Co., Ltd. (China) and were used without further purification. TCO-PEG_4_-NHS was purchased from Xi'an confluore Biological Technology Co., Ltd. Compound **3** was purchased from GL Biochem (Shanghai) Ltd. Column chromatography purification was performed on silica gel (54–74 μm, Qingdao Haiyang Chemical Co., Ltd., China). Anhydrous dichloromethane, anhydrous tetrahydrofuran (THF), anhydrous dimethyl sulfoxide (DMSO), anhydrous acetonitrile and anhydrous dimethylformamide (DMF) were purchased from Energy Chemical Co., Ltd. (China) and used without further drying.

### Synthesis of DOTA-PSMA-Tz

Full experimental details, including instrument information, characterization of the described compounds, and photoacoustic imaging method are provided in the Additional information.

### Radiochemistry

The radiolabeling procedure for [^68^Ga]Ga-PSMA-Tz and [^68^Ga]Ga-PSMA617 was performed as follows: 1 mL of ^68^GaCl_3_ (740 MBq in 0.1 M HCl) was diluted with 0.5 mL of sodium acetate (0.25 M in water, pH = 6.5) to generate ^68^GaCl_3_-sodium acetate solution (pH 4–4.5). The solution was then transferred to a vial containing approximately 0.03 μmol precursor dissolved in 10 μL of dimethyl sulfoxide (DMSO). The reaction mixture was subsequently heated for 15 min at 95 °C to form [^68^Ga]Ga-PSMA-Tz or [^68^Ga]Ga-PSMA617.

The radiolabeling procedure for [^177^Lu]Lu-PSMA-Tz was performed as follows: 740 MBq ^177^LuCl_3_ (in 0.04 M HCl) was diluted with 300 μL of 0.4 M ammonium acetate (pH = 5.6) and transferred into a vial containing 0.03 μmol precursor dissolved in 10 μL DMSO. The vial was subsequently heated for 30 min at 95 °C to form [^177^Lu]Lu-PSMA-Tz.

### Synthesis of Pd@Au-PEG-TCO

After the synthesis of Pd@Au-PEG_5000_-NH_2_ following the previously reported procedure [27], it was dissolved in 400 μL purified water (20 mg Pd@Au-PEG_5000_-NH_2_), and adjusted the pH to 8.0 by using 0.1 M Na_2_CO_3_. Then, 2 mg TCO-NHS dissolved in 25 μL DMSO was added to the above solution slowly. The mixture was stirred at 25 °C for 12 h to generate Pd@Au-PEG-TCO. After the reaction was completed, the mixture was centrifuged through ultrafiltration to remove unreacted TCO-NHS.

### Cell culture and xenograft models

22Rv1 cells were provided by the Cell Bank of the Chinese Academy of Sciences in Shanghai. The cells were cultured in Dulbecco’s modified Eagle's medium (DMEM, GIBCO), supplemented with 10% fetal bovine serum (FBS, GIBCO), 1% 100 μg/mL streptomycin and 100 IU/mL penicillin (MRC) at 37 °C in the humidified circumstance containing 5% CO_2_. Xenografts were implanted with 22Rv1 cells (1 × 10^6^) in the right axilla of BALB/c nude mice (18–20 g, 4–6 weeks, male). Animal experiments were approved by the Institutional Animal Care and Use Committee of Xiamen University.

### Cell uptake studies

The [^68^Ga]Ga-PSMA-Tz and [^68^Ga]Ga-PSMA617 were added to 22Rv1 cells respectively and incubated at 37 °C for 15, 30, 60, 90, and 120 min for cellular uptake studies. For blocking studies, additional PSMA617 (20 μg/well) was added for co-incubation. At the indicated time, the cells were washed, lysed, and collected for counts using an γ-counter. To validate the concept, cellular experiments were further divided into four groups. Firstly, the 22Rv1 cells were incubated with 100 μL of [^68^Ga]Ga-PSMA-Tz (37 kBq) (Groups I, III, and IV) or PBS (Group II) at 4 °C for 1 h. Subsequently, 100 μL of PBS, Pd@Au-PEG-TCO, Pd@Au-PEG-NH_2_, and Pd@Au-PEG-TCO were added to Groups I, II, III, and IV, respectively, and incubated for 5 min at 4 °C. Finally, 100 μL of [^68^Ga]Ga-PSMA-Tz were added to Groups I, III, IV (37 kBq) or II (74 kBq). After incubating at 4 °C for 1 h, the culture medium was removed, and the cells were washed twice with cold PBS (pH 7.4), and lysed with 1 mL of NaOH (0.1 M). The cell uptake of [^68^Ga]Ga-PSMA-Tz in Group I was designated as 100%.

### Cellular efflux assay

The cellular efflux assay was conducted by co-incubating 22Rv1 cells with [^68^Ga]Ga-PSMA-Tz (37 kBq/200 μL) at 37 °C for 1 h with or without Pd@Au-PEG-TCO pre-incubation for 1 h. Following the incubation, the culture medium was removed, and the cells were washed twice with cold PBS (pH 7.4) and then incubated with PBS for an additional 15, 30, 60, 90, and 120 min. Finally, the cells were lysed with 1 mL of NaOH (0.1 M). Subsequently, cell lysates were collected, and the radioactive counts were determined using an γ-counter.

### Saturation binding assays

For saturation binding assays, different concentrations of [^68^Ga]Ga-PSMA617 or [^68^Ga]Ga-PSMA-Tz were added to 22Rv1 cells with or without unlabeled PSMA617 (20 μg/well) pretreatment. After incubating at 37 °C for 1 h, the cells were washed, lysed, and collected for counts using an γ-counter to determine non-specific binding (NSB) and total binding (TB). Specific binding (SB) was derived and plotted against the concentrations of [^68^Ga]Ga-PSMA617 or [^68^Ga]Ga-PSMA-Tz to calculate the dissociation constant K_d_ using GraphPad Prism 7.0 software (San Diego, USA).

### MicroPET-CT imaging

The 22Rv1 tumor-bearing BALB/c mice (n = 3) were intravenously injected with 3.7–7.4 MBq of [^68^Ga]Ga-PSMA-Tz, and the subsequent scans of 5-min static PET and 7-min CT (80 kV, 50 μA) were performed at 15, 30, 60 and 120 min post-injection (p.i.) respectively with an Inveon PET-CT scanner under anesthesia using the mixture of 1.5% isoflurane with air to maintain spontaneous breathing during imaging. All PET images were reconstructed through the 2D/3D ordered-subset expectation–maximization (2D/3D OSEM) algorithm. The regions of interest (ROIs) were drawn to calculate the quantitative data (expressed as percentage injected dose per gram of tissue, %ID/g) and T/NT ratios of tissues. The blocking experiment was further conducted by intravenous (i.v.) co-injection of PSMA617 (2 mg/kg) and [^68^Ga]Ga-PSMA-Tz. For the proof-of-concept experiments, mice were randomly divided into four groups (n = 4/group). At 1 h after the first i.v. injection of [^68^Ga]Ga-PSMA-Tz, Groups II, III, and IV mice received 200 μg of Pd@Au-PEG-NH_2_, Pd@Au-PEG-TCO + PSMA617 (100 μg), and Pd@Au-PEG-TCO respectively through i.v. injection. Group I was set as control. At 24 h after the first injection, each mouse in all groups received a second i.v. injection of [^68^Ga]Ga-PSMA-Tz, followed by PET-CT imaging at 1 h p.i.

### Ultrafiltration assay

Ultrafiltration tubes with or without 20 μg of Pd@Au-PEG-TCO were added approximately 37 kBq [^68^Ga]Ga-PSMA-Tz solution in PBS. After incubating at 37 °C for 1 min, samples were centrifuged at 10,000 rpm for 10 min and were washed with water three times. The radioactive counts in the ultrafiltration tubes were measured using an γ-counter.

### Biodistribution studies

22Rv1 tumor-bearing BALB/c mice (n = 4) were intravenously injected with 1.48 MBq [^177^Lu]Lu-PSMA-Tz. The mice in Group ii were pretreated with 200 μg of Pd@Au-PEG-TCO at 24 h before the injection of [^177^Lu]Lu-PSMA-Tz. At indicated time points (4, 12, 24, 48 h), all mice were sacrificed and dissected. The organs/tissues of interest were collected, weighed, and counted by γ-counter to calculate the percentage of injected dose per gram (%ID/g, mean ± SD). For the biodistribution of Pd@Au-PEG-TCO, normal BALB/c mice (n = 4) were intravenously injected with 200 μg of Pd@Au-PEG-TCO. At 4, 12, 24, and 48 h p.i., mice were sacrificed and dissected to collect the organs/tissues of interest and weighed. Inductively coupled plasma mass spectrometry (ICP-MS) was used to measure the quantity of Au to calculate the percentage of injected dose per gram (%ID/g, mean ± SD).

### Statistical analysis

The statistical analyses were performed via the rigorous unpaired two-tailed Student's t-test using GraphPad Prism 8.0.2 software. All the data were expressed as mean ± SD. *P* < 0.05 was considered statistically significant. *P < 0.05, **P < 0.01, ***P < 0.001.

## Results and discussion

To precisely ensure effective signal amplification in tumors, we meticulously selected a tumor-targeting hydrophilic probe moiety and a nanoplate moiety for signal amplification, which demonstrate completely different metabolic behavior. The conjugation of Tz and TCO onto the probe and nanoplate moieties further allowed the click-mediated pre-targeting signal amplification in vivo. DOTA-PSMA-Tz was rationally designed as a model targeting probe that ideally incorporated three parts: the targeting moiety PSMA, the bifunctional chelator DOTA, and the bioorthogonal group Tz. DOTA-PSMA-Tz was synthesized by a straightforward route and characterized by ^1^H NMR, ^13^C NMR, MS, and HPLC, respectively (Additional file 1: Figures S1-S11 in the Additional file). [^68^Ga]Ga-PSMA-Tz was prepared in high radiochemical yield (89.6 ± 4.3%) with high radiochemical purity (> 98%), and suitable specific activity (∼9.25 GBq/μmol) using previously reported protocols (Additional file 1: Figs. 1A, S10). [^177^Lu]Lu-PSMA-Tz was also obtained with high radiochemical purity (> 97%) using the similar procedure (Additional file 1: Figure S11). [^68^Ga]Ga-PSMA-Tz demonstrated excellent in vitro stability within 2 h with no demetallation, in PBS and serum (Additional file 1: Figure S12). In blood and urine, [^68^Ga]Ga-PSMA-Tz existed mainly in the form of the parent compound at 60 min p.i. (Additional file 1: Figure S13). The excellent stability both in vitro and in vivo provided prerequisites to realize the click-mediated pre-targeted in vivo. [^68^Ga]Ga-PSMA-Tz revealed a nanomolar affinity for PSMA on human prostate carcinoma epithelial cell 22Rv1 cells (*K*_d_ = 16.40 ± 3.11 nM; n = 4), which has no significant difference (*P* > 0.05) to [^68^Ga]Ga-PSMA617 (*K*_d_ = 13.35 ± 3.35 nM, n = 4) (Additional file 1: Figure S14). In addition, cellular uptakes of [^68^Ga]Ga-PSMA617 and [^68^Ga]Ga-PSMA-Tz in 22Rv1 cells demonstrated a time-dependent increase reaching saturation after 60 min of incubation and both of the uptakes could be blocked by PSMA617, indicating the PSMA-specific uptakes in 22Rv1 cells (Additional file 1: Figure S15).

**Fig. 1:**
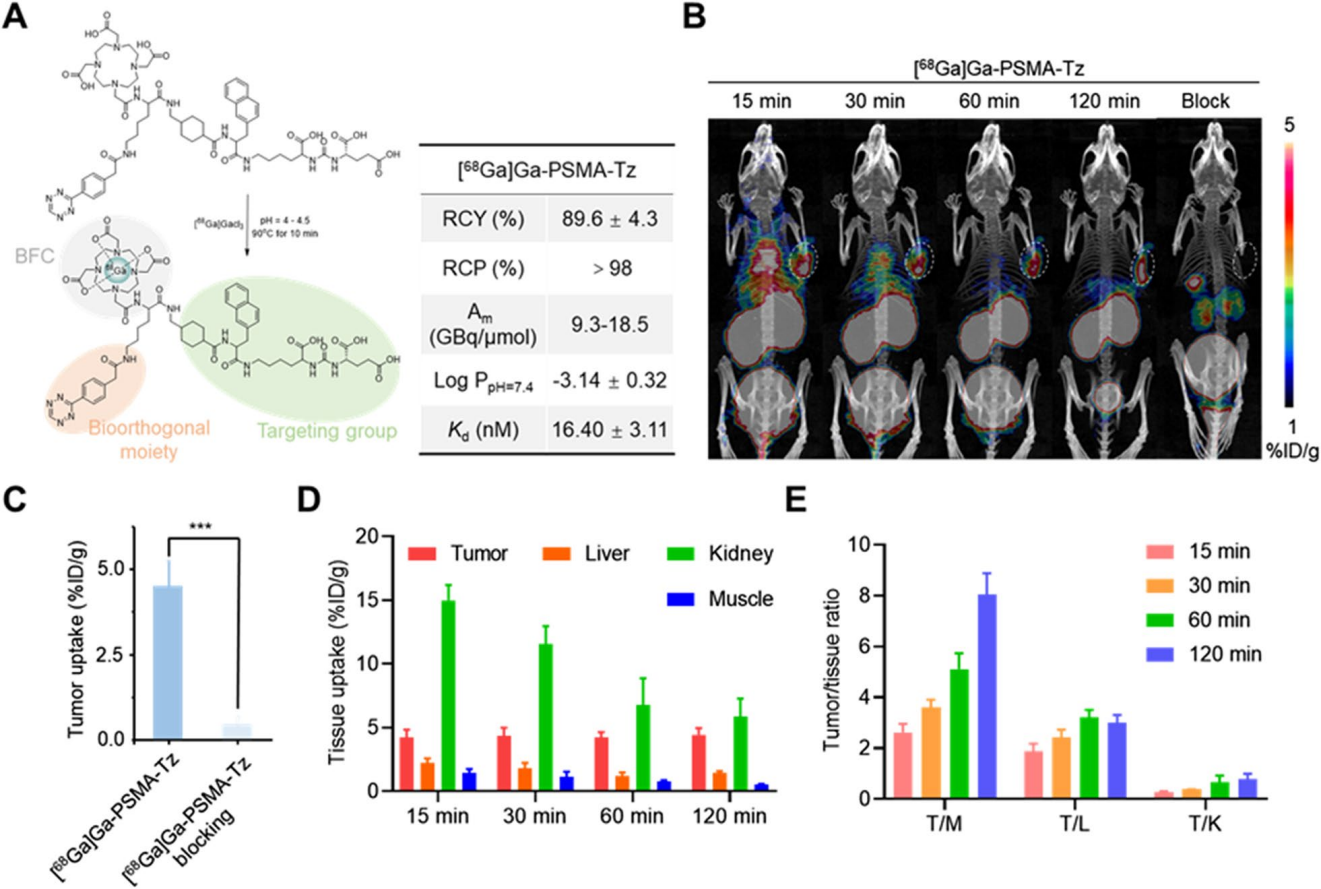
Validation of [^68^Ga]Ga-PSMA-Tz with PET imaging. **A** Radiosynthesis of [^68^Ga]Ga-PSMA-Tz. **B** MicroPET imaging of 22Rv1 xenograft models (n = 3) after injection of [^68^Ga]Ga-PSMA-Tz. Unlabeled PSMA617 was used as a blocking ligand. The tumor uptakes at 120 min p.i. (**C**), the tissue uptakes of main organs (**D**), and tumor/tissue ratios (**E**) were derived from the PET images

The in vivo PSMA-targeting ability and the imaging window of [^68^Ga]Ga-PSMA-Tz were studied by PET in 22Rv1 tumor-bearing mice. The tumor can be clearly visualized at 60 min p.i., and this high uptake of [^68^Ga]Ga-PSMA-Tz was almost completely blocked by co-injection of PSMA617 (Fig. 1B–C) (*P* < 0.001). Moreover, [^68^Ga]Ga-PSMA-Tz was cleared quickly from the kidneys (Fig. 1D), followed by a rapid accumulation of radioactivity in the bladder. The high T/NT ratios were obtained after 60 min injection (Fig. 1E). To wit, it is optimal to allow a 1–2 h imaging window for [^68^Ga]Ga-PSMA-Tz before administering the TCO-bearing signal amplification moiety.

**Fig. 2:**
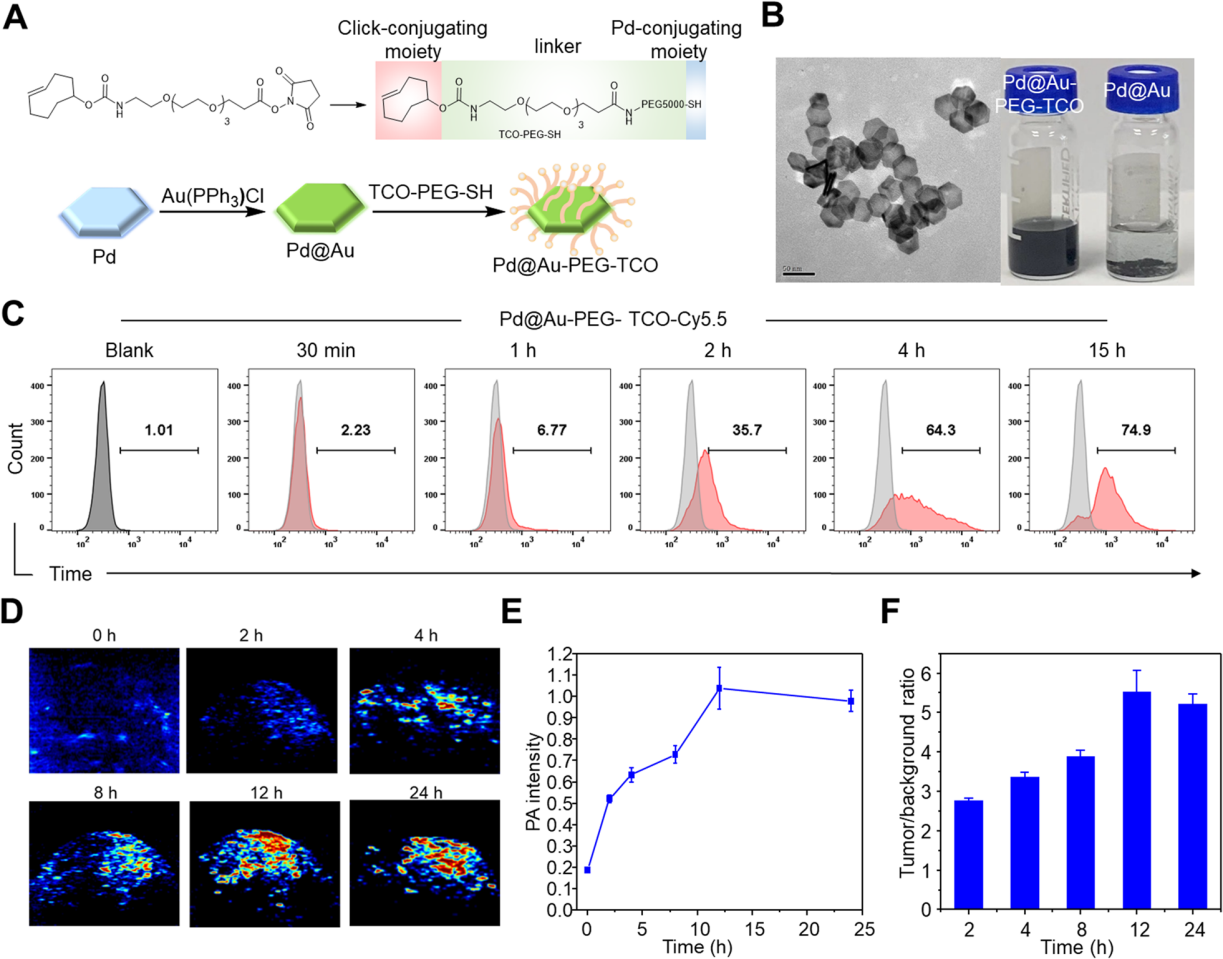
Preparation and evaluation of Pd@Au-PEG-TCO. **A** Synthesis of Pd@Au-PEG-TCO. **B** TEM image of Pd@Au-PEG-TCO. **C** The efficiency of cell uptake of Pd@Au-PEG-TCO-Cy5.5 after co-incubation with 22Rv1 cells. The PA images (**D**), time-intensity curves (**E**), and tumor/background ratios (**F**) of 22Rv1 tumor-bearing mice (n = 3) after injection of Pd@Au-PEG-TCO

The signal amplification module plays a key role in this proof-of-concept of the signal amplification strategy, which significantly accumulates in tumors and provides click reaction partners TCO for [^68^Ga]Ga-PSMA-Tz. According to our previous research, Pd@Au-PEG nanoplates accumulated in tumor sites via the EPR effect [27]. In addition, the large functional surface area and easy-to-control surface chemistry of nanoplates allow them to carry a sufficient number of TCO groups (Fig. 2A). Therefore, Pd@Au-PEG nanoplates, as ideal signal amplification carriers, with a mean diameter of ∼ 30 nm were prepared according to published protocols. To improve the dispersibility, TCO-modified long-chained PEG molecules were adopted to modify the surface of Pd@Au nanoplates and endow them with excellent dispersibility in saline even after 24 h incubation at room temperature, as compared to Pd@Au that form distinct precipitate, verified the applicability of PEGylation (Fig. 2B). A one-week incubation in the saline would not affect the inherent NIR absorption of Pd@Au-PEG-TCO, confirming its excellent in vitro stability (Additional file 1: Figure S16). As shown in the transmission electron microscopy (TEM) images, Pd@Au-PEG-TCO with a homogeneously ultrasmall size (∼30 nm) displayed a monodisperse state. Loading of 1.3 μmol/20 mg of the TCO-decorated Pd@Au-PEG-TCO was determined using Tz-NHS, whose absorbance at 531 nm was observed to measure the quantity of Tz-NHS pulled out of the solution by the Pd@Au-PEG-TCO.

Flow cytometry was performed to investigate the cellular uptake efficiency of the Cy5.5-labeled Pd@Au-PEG-TCO. The cell uptake increased over time and saturated after 4 h of incubation (up to 64.3%) (Fig. 2C). The biodistribution was performed to assess the biobehavioral of Pd@Au-PEG-TCO in normal mice. As expected, Pd@Au-PEG-TCO mainly accumulated in the liver and spleen, while mostly cleared from blood at 24 h p.i. The accumulations of Pd@Au-PEG-TCO in blood were measured to be 18.35 ± 1.22%ID/g, 9.33 ± 0.69%ID/g, 2.67 ± 0.23%ID/g, and 2.84 ± 0.60%ID/g at the 4, 12, 24 and 48 h p.i., respectively (Additional file 1: Figure S17). Due to its rapid blood clearance, Pd@Au-PEG-TCO exhibits relatively low uptake at the late time points of 24 and 48 h p.i., which is advantageous for minimizing the binding of subsequently administered [^177^Lu]Lu-PSMA-Tz in the blood pool.

For evaluating the retention of Pd@Au-PEG-TCO at the tumor site, the proven photoacoustic (PA) contrast effect of Pd@Au-PEG motivated us to use PA imaging to visualize the in vivo accumulation of Pd@Au-PEG-TCO in 22Rv1 tumor-bearing models [27]. As shown in Fig. 2D, the increased PA signal intensity over time in the tumor, indicated the gradual and significant accumulation of Pd@Au-PEG-TCO in the tumor. The PA signals enhanced more than five-fold after 12 h of Pd@Au-PEG-TCO injection (*P* < 0.001). Therefore, considering the time needed for sufficient tumor accumulation of Pd@Au-PEG-TCO, the time window for the third injection was determined to be 24 h.

**Fig. 3:**
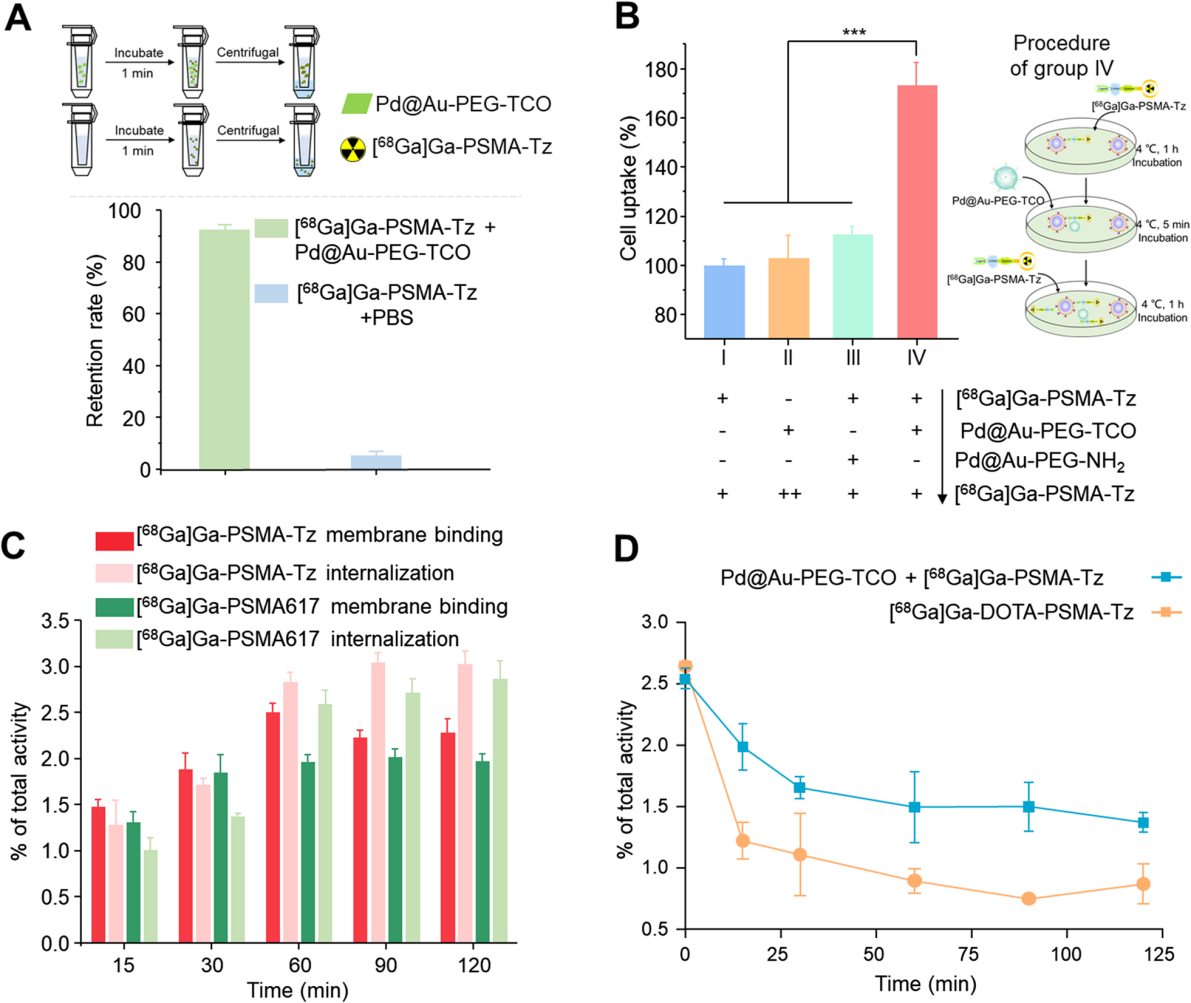
In vitro validation of click-mediated signal amplification. **A** The in vitro binding capacity of [^68^Ga]Ga-PSMA-Tz to Pd@Au-PEG-TCO was measured by ultrafiltration experiments. **B** The cell uptake assay of [^68^Ga]Ga-PSMA-Tz was performed with different procedures at 4 °C. The uptake of group I was defined as 100%. **C** Membrane binding and internalization of [^68^Ga]Ga-PSMA-Tz and [^68^Ga]Ga-PSMA617. **D** Time-dependent retention of [^68^Ga]Ga-PSMA-Tz in 22Rv1 cells with or without Pd@Au-PEG-TCO pre-incubation

For determining the click reaction ability of Pd@Au-PEG-TCO and [^68^Ga]Ga-PSMA-Tz in vitro, ultrafiltration assays were performed in PBS at room temperature. As expected, almost all [^68^Ga]Ga-PSMA-Tz (92 ± 4%) bound to Pd@Au-PEG-TCO, which can be attributed to the click reaction between the Tz group on the targeting probe ([^68^Ga]Ga-PSMA-Tz) and TCO on the amplify module (Pd@Au-PEG-TCO) (Fig. 3A). The abovementioned result confirmed that click-mediated reaction could effectively bond the two components under physiological conditions, and encouraged us to further explore the possibility of enhancing the tumor accumulation of the targeting probe using the amplification module. Under this assumption, cell uptake assays were performed with different sequential treatments at 4 °C, resulting in significantly inhibited cell internalization. As shown in Fig. 3B, the cellular uptake after twice [^68^Ga]Ga-PSMA-Tz (group I) incubation was defined as 100%. In group II, the lack of pre-incubation with [^68^Ga]Ga-PSMA-Tz resulted in the inability of Pd@Au-PEG-TCO to bind to the cell surface, consequently restricting the availability of additional binding sites for subsequent incubation with [^68^Ga]Ga-PSMA-Tz. Similarly, in group III, the signal amplification module is devoid of the TCO group, rendering it unable to provide additional binding sites for [^68^Ga]Ga-PSMA-Tz for signal amplification. A significant increase in cellular uptake was observed exclusively in group IV (*P* < 0.001), the success of which greatly depends on the integration of Pd@Au-PEG-TCO. The result demonstrated that Pd@Au-PEG-TCO can be effectively conjugated with the membrane-bound [^68^Ga]Ga-PSMA-Tz, providing additional binding sites for subsequent re-incubation of [^68^Ga]Ga-PSMA-Tz. Furthermore, the subsequently incubated [^68^Ga]Ga-PSMA-Tz exhibited the ability to bind both to the PSMA surface receptor and the pre-integrated Pd@Au-PEG-TCO linked to the cell membrane surface at the prior step (Fig. 3B).

**Fig. 4:**
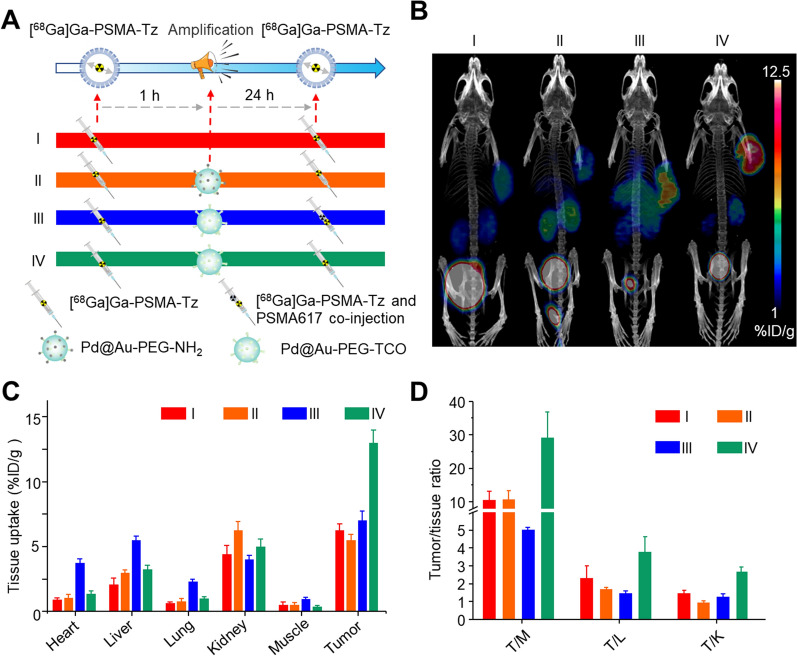
In vivo validation of click-mediated signal amplification. **A** Schematics of the procedures and timelines of signal amplification strategy in 22Rv1 tumor-bearing mice (n = 4). **B** MicroPET imaging of [^68^Ga]Ga-PSMA-Tz via the above procedures. Tissue uptakes (**C**) and T/NT ratios (**D**) were derived from the PET images

Considering that the cellular internalization of [^68^Ga]Ga-PSMA617 has been reported previously, we hereby investigated the internalization of [^68^Ga]Ga-PSMA-Tz and further verified its potential click reaction with the intracellular Pd@Au-PEG-TCO. An internalization study of [^68^Ga]Ga-PSMA-Tz was performed in 22Rv1 cells, revealing that approximately 2.5–3% of total activity was internalized after 1 h at 37 °C (Fig. 3C). Although the internalized [^68^Ga]Ga-PSMA-Tz and Pd@Au-PEG-TCO did not provide additional binding sites for the subsequent incubation of [^68^Ga]Ga-PSMA-Tz, it remains uncertain whether these components have the potential to react within the cell and further enhance the intracellular retention of [^68^Ga]Ga-PSMA-Tz. Therefore, the efflux assay of [^68^Ga]Ga-PSMA-Tz in 22Rv1 was performed*.* As shown in Fig. 3D, [^68^Ga]Ga-PSMA-Tz exhibited far faster clearance with the remaining activity of 32.9 ± 2.8% after 2 h of incubation. However, when in the case of pre-incubated with Pd@Au-PEG-TCO, the retention of [^68^Ga]Ga-PSMA-Tz in cells increased to 51.2 ± 1.0% after 2 h incubation (*P* < 0.001). These results demonstrated that the intracellular click reaction between [^68^Ga]Ga-PSMA-Tz and Pd@Au-PEG-TCO occurred, enhancing the retention of the radiotracer within the cells. In summary, both extracellular and intracellular click reactions occurred and played a key role in tumor retention and signal amplification through different mechanisms. Extracellular binding facilitates enhanced tumor uptake via a signal amplification module, whereas intracellular binding reduces the efflux of radioprobes. Both mechanisms are advantageous for increasing the accumulation of radiopharmaceuticals in tumors, which is conducive to radiotheranostic application. The proof-of-concept signal amplification strategy based on bioorthogonal click chemistry was further validated in vivo to demonstrate the utility of the novel concept of “click-to-amplify” selectively occurring in tumors via PET imaging (Fig. 4A). Notably, group IV produced dramatically higher tumoral uptake (11.5 ± 0.8%ID/g) than that of group I (5.5 ± 0.9%ID/g) without significantly increased uptakes in non-target organs. Meanwhile, the tumor-to-background uptake ratios of tumor-to-muscle (T/M), tumor-to-liver (T/L), and tumor-to-kidney (T/K) were 29.1 ± 7.8, 3.8 ± 0.9 and 2.3 ± 0.2 respectively in group IV, which were significantly higher than those of group I (T/M = 10.5 ± 2.6,* P* < 0.001; T/L = 2.3 ± 0.7, *P* < 0.05; T/K = 1.0 ± 0.2, *P* < 0.01, respectively). It is obviously suggested that signal amplification occurs only in tumors. For group II, mice treated with Pd@Au-PEG-NH_2_ nanoparticles without TCO modification showed no significant increase in tumor uptake (5.7 ± 0.5%ID/g). These results confirmed that the signal amplification at the tumor site was mediated by a bioorthogonal click chemistry reaction between the TCO and Tz groups. To elaborate on the dramatically increased tumoral uptake contributed by Pd@Au-PEG-TCO, 100 μg of PSMA617 was co-injected with [^68^Ga]Ga-PSMA-Tz to block the PSMA-mediated uptake while retaining the signal amplification function of Pd@Au-PEG-TCO, as designed in group III. Compared to group IV, group III showed a decreased tumor uptake value of 6.8 ± 0.5%ID/g (*P* < 0.001). Considering the full blockage effect of PSMA on tumor uptake of [^68^Ga]Ga-PSMA-Tz (Fig. 1B), it can be concluded the tumor uptake in group III relies predominantly on the Tz-TCO mediated click reaction. Simultaneously, the tumor uptake observed in Groups II and III registered average values of 5.7%ID/g and 6.8%ID/g, culminating in a cumulative total of 12.5%ID/g. This confluence of values, juxtaposed against the uptake of 11.5%ID/g in Group IV, underscores a lack of substantial distinction. The data clearly show that both the PSMA and signal amplification modules play critical roles in the strategy for the enhanced uptake of [^68^Ga]Ga-PSMA-Tz (Fig. 4B–D).

**Fig. 5:**
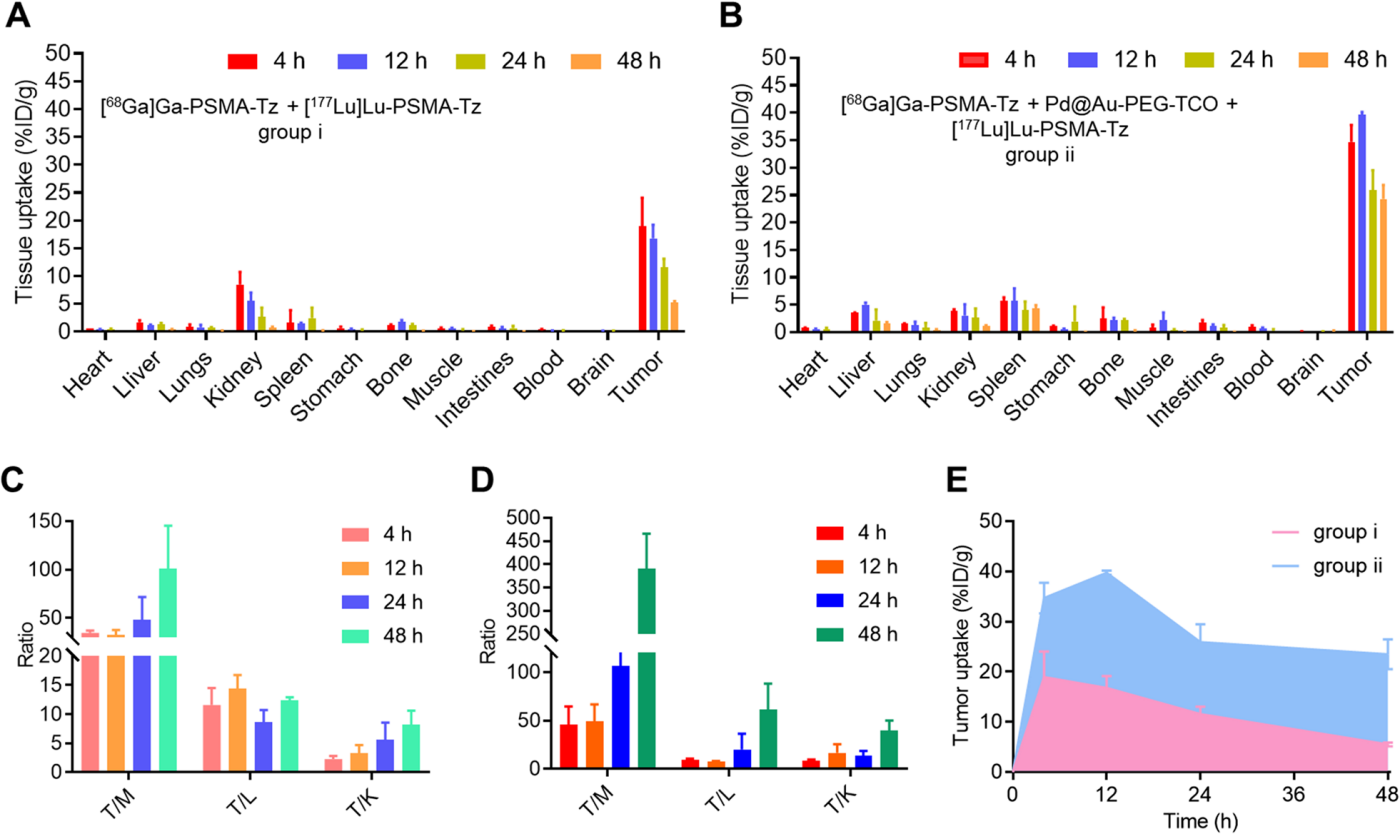
Ex vivo biodistribution of [^177^Lu]Lu-PSMA-Tz in 22Rv1 tumor-bearing mice (n = 4) at 48 h p.i. without (Group i) (**A**) or with (Group ii) (**B**) the pre-injection of Pd@Au-PEG-TCO. The tumor-to-muscle (T/M), tumor-to-liver (T/L), and tumor-to-kidney (T/K) ratios of Group i (**C**) and Group ii (**D**) were calculated from the results of biodistribution. **E** The AUCs of tumor uptake for groups i and ii were calculated and compared

Given the inherent compatibility and DOTA-binding efficiency, ^68^Ga and ^177^Lu were selected as a “theranostic pair” for this study. Specifically, ^68^Ga labeled radiotracers are well-suited for PET imaging, providing essential information on biodistribution and receptor abundance while guiding dosimetry for ^177^Lu; ^177^Lu, whereas, is a widely used radioisotope of choice for TRT. Therefore, it is reasonable to use PET with [^68^Ga]Ga-PSMA-Tz as a guide for the TRT of [^177^Lu]Lu-PSMA-Tz. After diagnosis using [^68^Ga]Ga-PSMA-Tz PET, biodistribution studies of [^177^Lu]Lu-PSMA-Tz (Group i) and the complex in signal amplification strategy (Group ii) were performed in 22Rv1 tumor-bearing mice (Fig. 5A, B). Tumor uptake reached 34.70 ± 3.06%ID/g at 4 h p.i. in Group ii, which was approximately two-fold higher in comparison to that of Group i (18.95 ± 5.11%ID/g) (*P* < 0.001). The significantly increased tumor uptake can be attributed to the Pd@Au-PEG-TCO, which offers a greater number of binding sites for [^177^Lu]Lu-PSMA-Tz. Moreover, tumor uptakes of [^177^Lu]Lu-PSMA-Tz in Group i decreased gradually over the p.i. time (18.95 ± 5.11%ID/g at 4 h, 16.79 ± 2.41%ID/g at 12 h, 11.58 ± 1.52%ID/g at 24 h and 5.19 ± 0.26%ID/g at 48 h), only approximately 27% of tumor uptake was retained at 48 h when compared to that of 4 h p.i. Compared to Group i, [^177^Lu]Lu-PSMA-Tz in Group ii, with prior administration of Pd@Au-PEG-TCO, exhibited a significantly slower clearance from the tumor over 48 h (from 34.70 ± 3.06% ID/g at 4 h p.i. to 24.33 ± 2.53% ID/g at 48 h p.i.), approximately 70% of tumor uptake was retained at 48 h when compared to that of 4 h p.i. This indicated that a click reaction occurred between [^177^Lu]Lu-PSMA-Tz and Pd@Au-PEG-TCO, resulting in prolonged tumor retention of [^177^Lu]Lu-PSMA-Tz, which was consistent with the findings in the cell efflux assay. Owing to the significantly enhanced tumor uptake and prolonged tumor retention, Group ii demonstrates notable advantages in terms of T/NT ratio compared to Group i at 48 h. In Group ii, the tumor-to-background uptake ratios of T/M, T/L, and T/K were 391.14 ± 74.74, 61.08 ± 27.13 and 39.73 ± 10.30, respectively, which were significantly higher than that of Group i (T/M = 100.87 ± 44.43, *P* < 0.01; T/L = 12.34 ± 0.57, *P* < 0.05; and T/K = 8.22 ± 2.37, *P* < 0.01, respectively) (Fig. 5C, D).

The area under the curve (AUC) provides a measurement of the actual exposure of the body to the radioligand following administration. A larger AUC for the tumor indicates an extended duration of the radioligand within the tumor microenvironment and a slower decrease in drug activity, implying enhanced therapeutic efficacy. Comparing AUCs makes the selection of the strategy with the highest tumor exposure and bioavailability possible. AUCs of tumor and kidneys were calculated for [^177^Lu]Lu-PSMA-Tz in groups i and ii using biodistribution results in 22Rv1 tumor mice. As shown in Fig. 5E, Group ii showed significantly higher tumor AUC (1354.17 ± 61.73) compared to that in Group i (556.40 ± 35.48, *P* < 0.001), indicating almost 2.5-fold as high as tumor exposure level and higher bioavailability of [^177^Lu]Lu-PSMA-Tz in Group ii over Group i. Meanwhile, [^177^Lu]Lu-PSMA-Tz in Group ii showed significantly lower kidney AUC (117.86 ± 16.75) compared to that in Group i (162.20 ± 26.02, *P* < 0.05). Collectively, the click-mediated signal amplification module was able to produce dramatic improvement in tumor uptake and enhance the retention time of radioactivity in the tumor site, which might lead to a prominent antitumor effect of radionuclide therapy in vivo.

In summary, this proof-of-concept study demonstrated that the signal amplification strategy is a highly effective platform for tumor radiotheranostic application. Here we innovatively proposed a three-step protocol, designed and obtained the match of radiolabeled targeting probes with Tz-modification ([^68^Ga]Ga-PSMA-Tz and [^177^Lu]Lu-PSMA-Tz) and TCO-modified amplification module Pd@Au-PEG-TCO. We validated the bioorthogonal click chemistry in vitro and in vivo. In PSMA-positive prostate cancer, we successfully improved the tumor uptake to about five-fold higher and prolonged the tumor retention effectively. Based on these promising results, we believe that this approach is worth investigating further and finally providing an intelligent paradigm for cancer radiotheranostics.

### Supplementary Information

**Additional file 1: Scheme S1.** The synthetic route of DOTA-PSMA-Tz (**6**): (i) a) Hydrazine hydrate, S, EtOH, 50 °C, 24 h; b) NaNO_2_, AcOH, RT, 1 h; (ii)* N*, *N*'-Disuccinimidyl carbonate, DIPEA, CH_2_Cl_2_, RT, 8 h; (iii) DOTA-NHS, TEA, DMSO, 8 h; (iv) Hydrazine hydrate (85%)/H_2_O (1:50), RT, 2 h; (v) TEA, DMSO, 12 h. **Figure S1.** ^1^H NMR (400 MHz, DMSO) spectrum of **1**. **Figure S2.** ^13^C NMR (101 MHz, DMSO) spectrum of **1**. **Figure S3 **^1^H NMR (400 MHz, CDCl_3_) spectrum of **2**. **Figure S4.** ^13^C NMR (101 MHz, CDCl_3_) spectrum of **2**. **Figure S5.** Mass spectrum of **4**. **Figure S6.** Mass spectrum of **5**. **Figure S7.** Mass spectrum of DOTA-PSMA-Tz. **Figure S8.** ^1^H NMR (400 MHz, DMSO) spectrum of DOTA-PSMA-Tz. **Figure S9.** ^13^C NMR (101 MHz, DMSO) spectrum of DOTA-PSMA-Tz. **Figure S10.** Analytical HPLC chromatogram of [^68^Ga]Ga-PSMA-Tz and DOTA-PSMA-Tz. **Figure S11.** Analytical HPLC chromatogram of [^177^Lu]Lu-PSMA-Tz and DOTA-PSMA-Tz. **Figure S12**. Stabilities of [^68^Ga]Ga-PSMA-Tz in PBS (**a**) and serum (**b**). **Figure S13.** *In vivo* metabolic stability of [^68^Ga]Ga-PSMA-Tz in healthy C57BL/6 mice through radio-HPLC analysis of blood (a) and urine (b). **Figure S14.** Saturation binding assay of [^68^Ga]Ga-PSMA617 and [^68^Ga]Ga-PSMA-Tz. All data are shown as mean ± SD (*n* = 4). **Figure S15.** The specific uptake of [^68^Ga]Ga-PSMA-Tz and [^68^Ga]Ga-PSMA617. All data are shown as mean ± SD (*n* = 4). **Figure S16.** The absorption spectrum of Pd@Au-PEG-TCO after incubation in the saline for 0, 1, 3, 5, and 7 d, respectively. **Figure S17.** Biodistribution of Pd@Au-PEG in different organs at different times. All data are shown as mean ± SD (*n* = 4).

## Data Availability

The datasets generated and/or analyzed during the current study are available in the article and Supporting Information.
