# Supplementary material for: A proof-of-concept study on bioorthogonal-based pretargeting and signal amplify radiotheranostic strategy
Source: J Nanobiotechnology. 2024 Mar 10;22:101. doi: 10.1186/s12951-024-02312-y (PMC10926607; doi:10.1186/s12951-024-02312-y)
Supplement: Supplementary file 1 — Additional file 1: Scheme S1. The synthetic route of DOTA-PSMA-Tz (6): (i) a) Hydrazine hydrate, S, EtOH, 50 °C, 24 h; b) NaNO2, AcOH, RT, 1 h; (ii) N, N'-Disuccinimidyl carbonate, DIPEA, CH2Cl2, RT, 8 h; (iii) DOTA-NHS, TEA, DMSO, 8 h; (iv) Hydrazine hydrate (85%)/H2O (1:50), RT, 2 h; (v) TEA, DMSO, 12 h. Figure S1. 1H NMR (400 MHz, DMSO) spectrum of 1. Figure S2. 13C NMR (101 MHz, DMSO) spectrum of 1. Figure S3 1H NMR (400 MHz, CDCl3) spectrum of 2. Figure S4. 13C NMR (101 MHz, CDCl3) spectrum of 2. Figure S5. Mass spectrum of 4. Figure S6. Mass spectrum of 5. Figure S7. Mass spectrum of DOTA-PSMA-Tz. Figure S8. 1H NMR (400 MHz, DMSO) spectrum of DOTA-PSMA-Tz. Figure S9. 13C NMR (101 MHz, DMSO) spectrum of DOTA-PSMA-Tz. Figure S10. Analytical HPLC chromatogram of [68Ga]Ga-PSMA-Tz and DOTA-PSMA-Tz. Figure S11. Analytical HPLC chromatogram of [177Lu]Lu-PSMA-Tz and DOTA-PSMA-Tz. Figure S12. Stabilities of [68Ga]Ga-PSMA-Tz in PBS (a) and serum (b). Figure S13. In vivo metabolic stability of [68Ga]Ga-PSMA-Tz in healthy C57BL/6 mice through radio-HPLC analysis of blood (a) and urine (b). Figure S14. Saturation binding assay of [68Ga]Ga-PSMA617 and [68Ga]Ga-PSMA-Tz. All data are shown as mean ± SD (n = 4). Figure S15. The specific uptake of [68Ga]Ga-PSMA-Tz and [68Ga]Ga-PSMA617. All data are shown as mean ± SD (n = 4). Figure S16. The absorption spectrum of Pd@Au-PEG-TCO after incubation in the saline for 0, 1, 3, 5, and 7 d, respectively. Figure S17. Biodistribution of Pd@Au-PEG in different organs at different times. All data are shown as mean ± SD (n = 4). [file 12951_2024_2312_MOESM1_ESM.docx]

**A Proof-of-Concept Study on Bioorthogonal-based Pretargeting and Signal Amplify Radiotheranostic Strategy**

Hongzhang Yang,^#[1]^ Xinying Zeng,^#[1]^ Jia Liu,^#[1]^ Jingchao Li,^[3]^ Yun Li,^[1]^ Qinglin Zhang,^[1]^ Linlin Shu,^[1]^ Huanhuan Liu,^[1]^ Xueqi Wang,^[1]^ Yuanyuan Liang,^[1]^ Ji Hu,^[4]^ Lumei Huang,*^[1]^ Zhide Guo,*^[1]^ and Xianzhong Zhang*^[2]^

[1] State Key Laboratory of Vaccines for Infectious Diseases, Center for Molecular Imaging and Translational Medicine, Xiang An Biomedicine Laboratory, School of Public Health, Xiamen University. Xiamen 361102, China.
E-mail: gzd666888@xmu.edu.cn, E-mail: huanglmchemistry@126.com

| **Contents** | **Page** |
| --- | --- |
| General information | p. S2 |
| Synthesis | p. S3 |
| Supplementary NMR and mass spectra | p. S6 |
| Radiochemistry  Stability | p. S10  p. S11 |
| Log *D* | p. S12 |
| Saturation binding assays | p. S13 |
| Time-dependent cell uptake | p. S13 |
| In vitro stability of Pd@Au-PEG-TCO | p. S14 |
| Biodistribution of Pd@Au-PEG-TCO | p. S14 |
| PA imaging | p. S15 |

[2] Theranostics and Translational Research Center, Institute of Clinical Medicine & Department of Nuclear Medicine, Peking Union Medical College Hospital, Chinese Academy of Medical Sciences & Peking Union Medical College. Beijing 100730, China.

Email: zhangxzh@pumch.cn

[3] PET Center, Department of Nuclear Medicine, The First Affiliated Hospital, School of Medicine, Zhejiang University. 79 Qingchun Road, Hangzhou 310003, China.

[4] HTA Co., Ltd., No. 1 Sanqiang Road, Fangshan District, Beijing 102413, China

Correspondence and requests for materials should be addressed to X. Z. ([zhangxzh@pumch.cn](mailto:zhangxzh@pumch.cn)).

**Table of Contents**

Additional Information

1. **General information**

## General reagent information

All the reagents we used in the synthesis and biology experiment were purchased from Energy Chemical Co., Ltd. (China) or J&K Co., Ltd. (China) and were used without further purification. TCO-PEG4-NHS was purchased from Xi'an confluore Biological Technology Co., Ltd. Compound 3 was purchased from GL Biochem (Shanghai) Ltd. Column chromatography purification was performed on silica gel (54–74 μm, Qingdao Haiyang Chemical Co., Ltd., China). Anhydrous dichloromethane, anhydrous tetrahydrofuran (THF), anhydrous dimethyl sulfoxide (DMSO), anhydrous acetonitrile and anhydrous dimethylformamide (DMF) were purchased from Energy Chemical Co., Ltd. (China) and used without further drying.

## General instrument information

Proton-1 and carbon-13 nuclear magnetic resonance (^1^H, ^13^C NMR) spectra were recorded on an AS 400 MHz NMR spectrometer (ZhongKeNiuJin, China, ^1^H NMR at 400 MHz, ^13^C NMR at 100 MHz). Mass spectrometry was acquired on Waters Xevo G2-XS Tof (Waters, U.S.A). High-performance liquid chromatography (HPLC) analysis and purification were performed on a Thermo Ultimate 3000 equipped with a Flow Count 3200 NaI/PMT γ-radiation scintillation detector (Bioscan, U.S.A). MicroPET-CT imaging was performed on a Siemens Inveon scanner (Siemens, U.S.A), and the radioactive samples (cells or tissues) were measured by a Gamma counter (WIZARD 2480, PerkinElmer, U.S.A). Photoacoustic (PA) imaging was achieved through Visualsonics LAZR-X Vevo (Fujifilm, Japan).

## General chemical analysis information

Thin layer chromatography (TLC) was performed on TLC Silica gel 60 F254 aluminum sheets (Merck, Germany), and visualized with short wave UV light (254 nm) or iodine staining. Tetramethylsilane (TMS) was sometimes used as an internal standard for ^1^H NMR, and all the chemical shifts were reported as δ values relative to the internal TMS. Chemical shifts for protons were reported in parts per million (ppm) downfield from TMS and are referenced to residual protium in the solvent (^1^H NMR: CDCl_3_ at 7.26 ppm, D_2_O at 4.79 ppm, MeOD at 3.31 ppm, and DMSO-*d_6_* at 2.50 ppm). Chemical shifts for ^13^C signals are referenced to the carbon resonances of the solvent peak (^13^C NMR: CDCl_3_ at 77.16 ppm, MeOD at 49.00 ppm, and DMSO-*d_6_* at 39.52 ppm). Multiplicity is defined by s (singlet), d (doublet), t (triplet), and m (multiplet). The coupling constants were reported in Hertz (Hz).

1. **Synthesis**

**Scheme S1**. The synthetic route of DOTA-PSMA-Tz (**6**): (i) a) Hydrazine hydrate, S, EtOH, 50 °C, 24 h; b) NaNO_2_, AcOH, RT, 1 h; (ii) *N*, *N*'-Disuccinimidyl carbonate, DIPEA, CH_2_Cl_2_, RT, 8 h; (iii) DOTA-NHS, TEA, DMSO, 8 h; (iv) Hydrazine hydrate (85%)/H_2_O (1:50), RT, 2 h; (v) TEA, DMSO, 12 h.

**2-(4-(1,2,4,5-Tetrazin-3-yl)phenyl)acetic acid (1)**

4-Cyanophenylacetic acid (2 mmol, 322 mg), CH_2_Cl_2_ (2 mmol, 170 mg), sulfur (4 mmol, 64 mg), and ethanol (2 mL) were mixed in a 30 mL reaction tube. 85% Hydrazine monohydrate (16 mmol, 0.8 ml) was added slowly with stirring afterward. The vessel was sealed and the reaction mixture was heated to 50 °C for 24 hours. Then 3 mL of CH_2_Cl_2_ and sodium nitrite (20 mmol, 1.4 g) in 10 mL of H_2_O were added to the mixture. Excess acetic acid (120 mmol, 6.82 mL) was then added slowly during which the solution turned bright red. The reaction mixture was extracted with dichloromethane. The organic phase was dried over anhydrous magnesium sulfate (MgSO_4_), filtered and concentrated under reduced pressure. The resulting residue was purified using silica gel chromatography.

^1^H NMR (400 MHz, DMSO): δ 10.60 (s, 1H), 8.48 (d, *J* = 8.1 Hz, 2H), 7.60 (d, *J* = 8.1 Hz, 2H), 3.77 (s, 2H).

^13^C NMR (101 MHz, DMSO): δ 172.72 (s), 165.92 (s), 158.62 (s), 140.75 (s), 131.05 (s), 128.31 (s).

**2,5-Dioxopyrrolidin-1-yl 2-(4-(1,2,4,5-tetrazin-3-yl)phenyl)acetate (2)**

To a stirred solution of tetrazine acid **1** (216 mg, 1 mmol) in CH_2_Cl_2_ (10 mL) were sequentially added *N*,*N*'-Disuccinimidyl carbonate (300 mg, 1.2 mmol) and DIPEA (250 mg, 2 mmol). The resultant mixture was stirred at 25 °C for 8 h before it was quenched with water. The organic layer was washed with brine and dried over anhydrous Na_2_SO_4_. After filtration and removal of the solvent under vacuum, The resulting residue was purified using silica gel chromatography.

^1^H NMR (400 MHz, CDCl_3_): δ 10.16 (s, 1H), 8.58 (d, *J* = 8.4 Hz, 2H), 7.54 (d, *J* = 8.4 Hz, 2H), 4.00 (s, 2H), 2.79 (s, 4H).

^13^C NMR (101 MHz, CDCl_3_): δ 168.87 (s), 166.16 (s), 157.86 (s), 136.73 (s), 131.22 (s), 130.40 (s), 128.78 (s), 37.63 (s), 25.59 (s).

**Synthesis of compound 4**

The mixture of **3** (7 mg, 5 μmol), DOTA-NHS (4.6 mg, 6 μmol), and 5 μL of triethylamine in 500 μL DMSO was shaken in an oscillator for 8 h at RT. The product was isolated using semi-preparative HPLC. C-18 reversed-phase column (4.6 × 250 mm, 5 μm, 120 Å, Thermo), using a mobile phase of 0.1 % TFA in water (A) and 0.1 % TFA in acetonitrile (B) as follows: 5 % B gradients to 95 % B at 30 min with a flow rate of 3 mL/min.

MS (ESI) calculated for C_65_H_96_N_11_O_19_ ([M+H]^+^): requires 1334.68, found 1334.69.

MS (ESI) calculated for C_65_H_97_N_11_O_19_ ([M+2H]^2+^/2): requires 667.84 found 667.85.

**Synthesis of 5**

Hydrazine monohydrate (85%, 7 μL) was sequentially added to a stirred solution of compound **4** (2 mg) in H_2_O (200 μL). The resultant mixture was stirred at room temperature for 2 h. The product was isolated using semi-preparative HPLC. C-18 reversed-phase column (4.6 × 250 mm, 5 μm, 120 Å, Thermo), using a mobile phase of 0.1 % TFA in water (A) and 0.1 % TFA in acetonitrile (B) as follows: 5 % B gradients to 95 % B at 30 min with a flow rate of 3 mL/min.

MS (ESI) calculated for C_55_H_84_N_11_O_17_ ([M+H]^+^): requires 1170.60, found 1170.60.

MS (ESI) calculated for C_55_H_85_N_11_O_17_ ([M+2H]^2+^/2): requires 585.80, found 585.81.

**Synthesis of DOTA-PSMA-Tz (6)**

The mixture of **5** (1.16 mg, 1 μmol), **2** (0.375 mg, 1.2 μmol), and 1 μL of triethylamine in 200 μL DMSO was shaken in an oscillator for 8 h at RT. The product was isolated using semi-preparative HPLC and lyophilized to give a pink solid product compound **6** (0.75 mg, yield 54.87%). C-18 reversed-phase column (4.6 × 250 mm, 5 μm, 120 Å, Thermo), using a mobile phase of 0.1 % TFA in water (A) and 0.1 % TFA in acetonitrile (B) as follows: 5 % B gradients to 95 % B at 30 min with a flow rate of 3 mL/min.

MS (ESI) calculated for C_65_H_90_N_15_O_18_ ([M+H]^+^): requires 1368.6, found 1368.4

MS (ESI) calculated for C_65_H_91_N_15_O_18_ ([M+2H]^2+^/2): requires 684.8, found 684.7

^1^H NMR (400 MHz, DMSO) δ 10.59 (s, 1H), 8.46 (d, *J* = 8.0 Hz, 4H), 8.26 (d, *J* = 17.1 Hz, 2H), 8.16 – 7.92 (m, 3H), 7.92 – 7.26 (m, 16H), 6.54 – 6.21 (m, 2H), 4.71 – 4.17 (m, 3H), 4.08 (ddd, *J* = 19.9, 13.1, 7.5 Hz, 5H), 3.53 (dd, *J* = 35.2, 20.3 Hz, 17H), 3.41 – 2.61 (m, 53H), 2.57 (s, 4H), 2.42 – 2.18 (m, 4H), 2.17 – 2.02 (m, 3H), 2.00 – 1.82 (m, 2H), 1.83 – 0.93 (m, 40H), 0.84 (t, *J* = 17.2 Hz, 2H).

^13^C NMR (101 MHz, DMSO) δ 175.48 (s), 175.06 (s), 174.70 (s), 174.26 (s), 172.04 (s), 171.71 (d, J = 39.6 Hz), 170.71 (s), 170.33 (m), 165.92 (s), 158.52 (d, J = 8.7 Hz), 157.77 (s), 142.44 (s), 136.25 (s), 133.32 (s), 132.20 (s), 130.60 (s), 130.38 (s), 128.24 (d, J = 23.3 Hz), 128.07 – 128.00 (m), 127.81 (m), 126.35 (s), 125.76 (s), 52.78 (s), 52.25 – 51.76 (m), 40.84 (s), 39.09 (s), 29.14 (s).

1. **Additional NMR and Mass spectra**


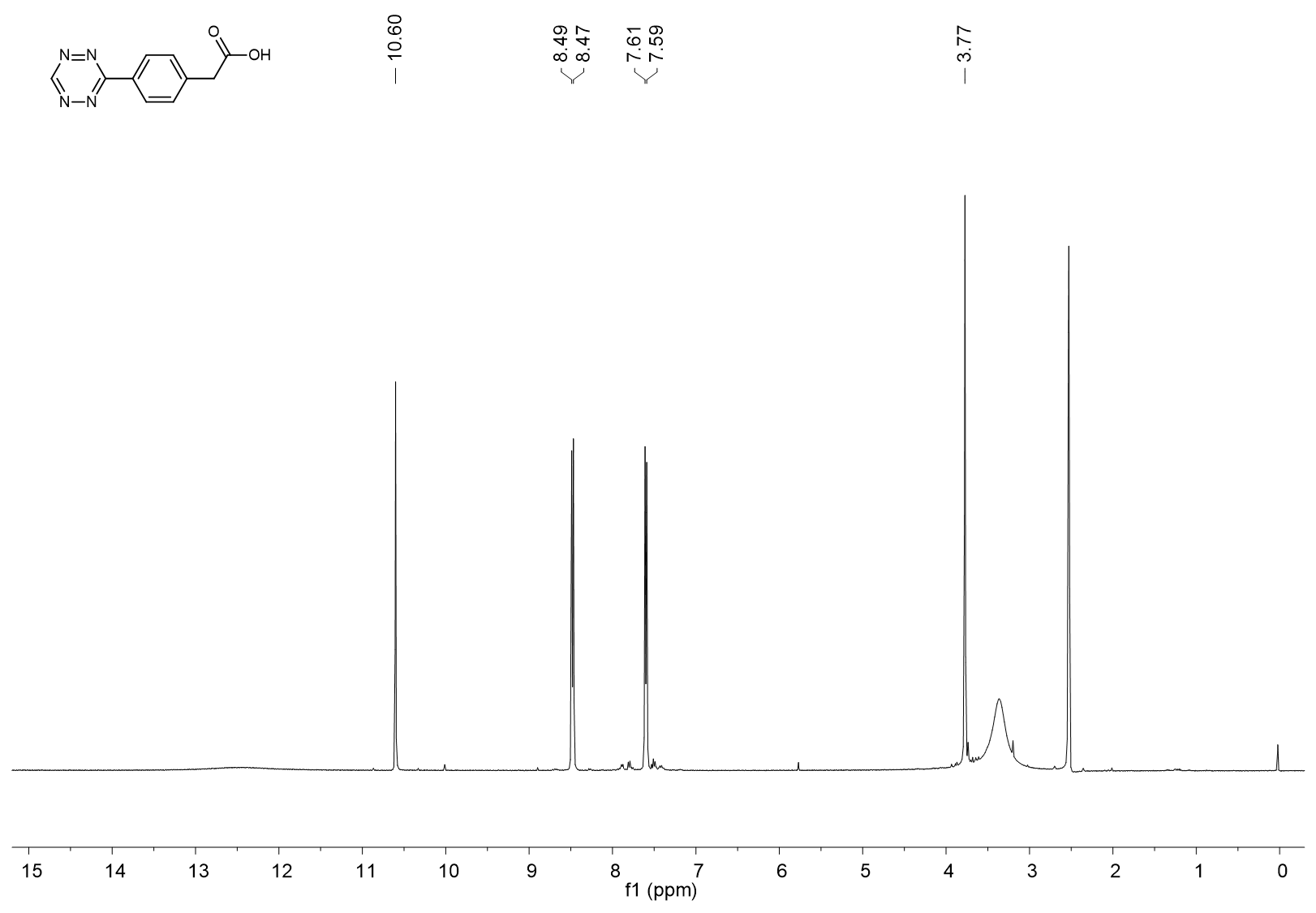


**Figure S1** ^1^H NMR (400 MHz, DMSO) spectrum of **1**.


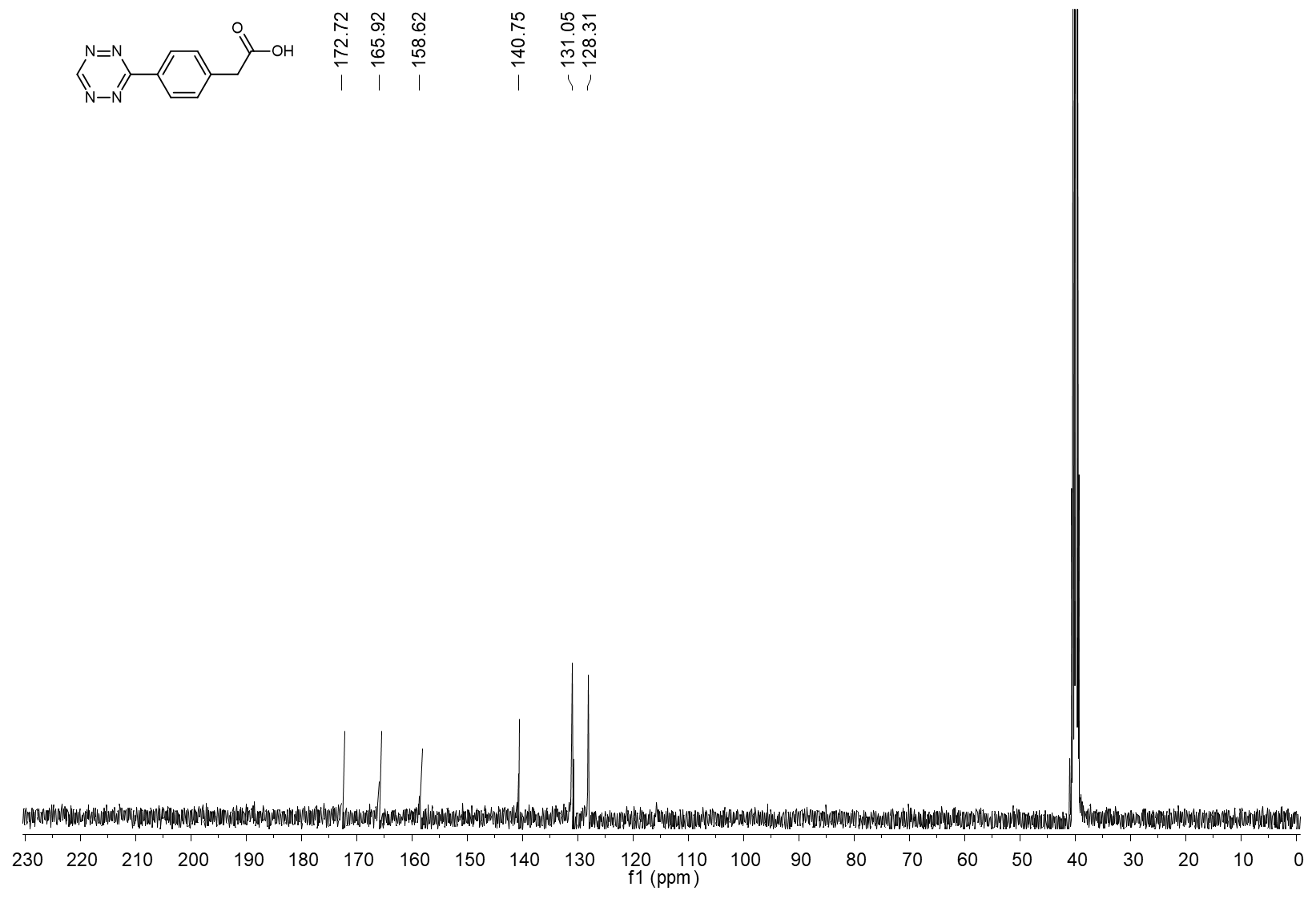


**Figure S2** ^13^C NMR (101 MHz, DMSO) spectrum of **1**.


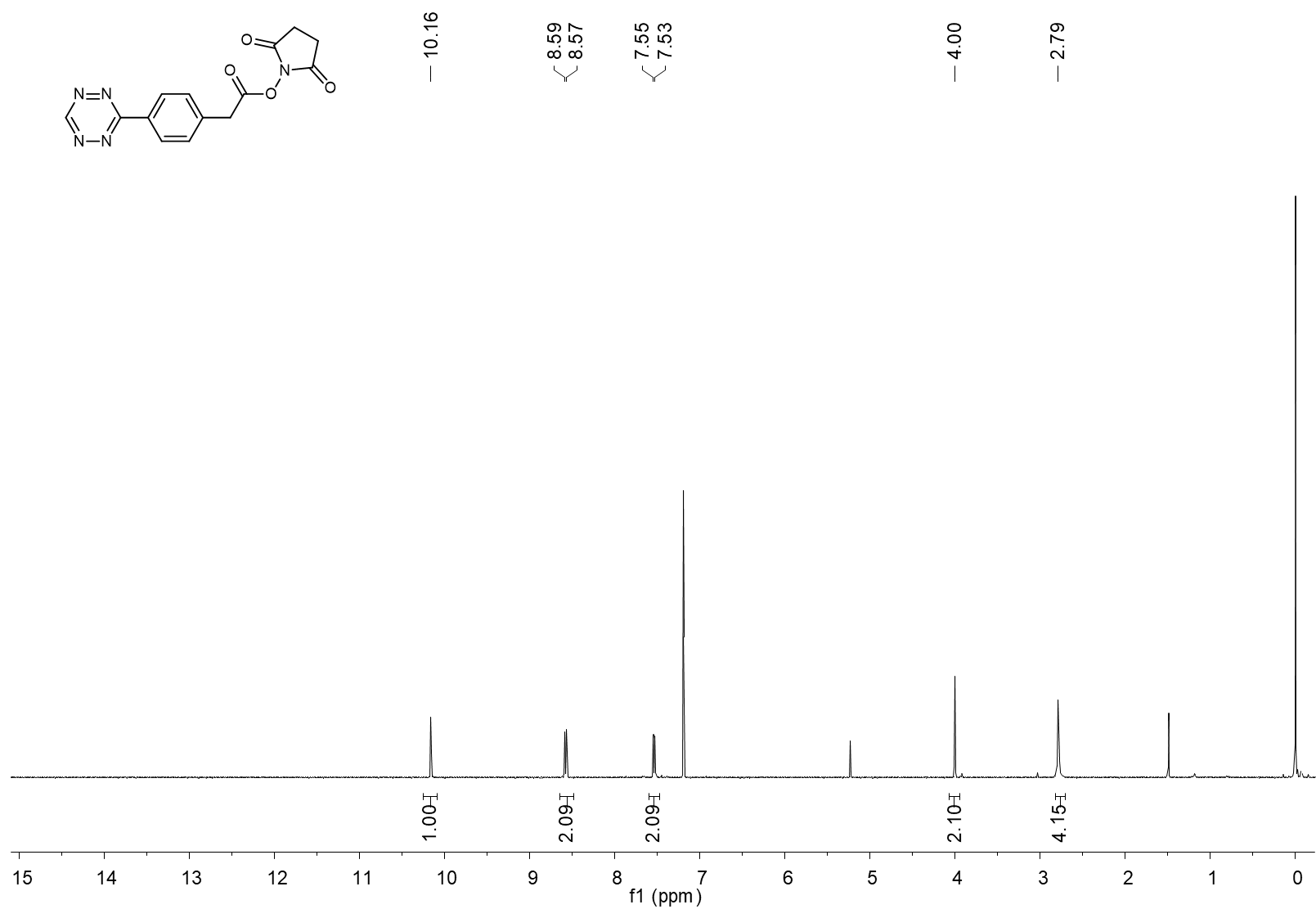


**Figure S3** ^1^H NMR (400 MHz, CDCl_3_) spectrum of **2**.


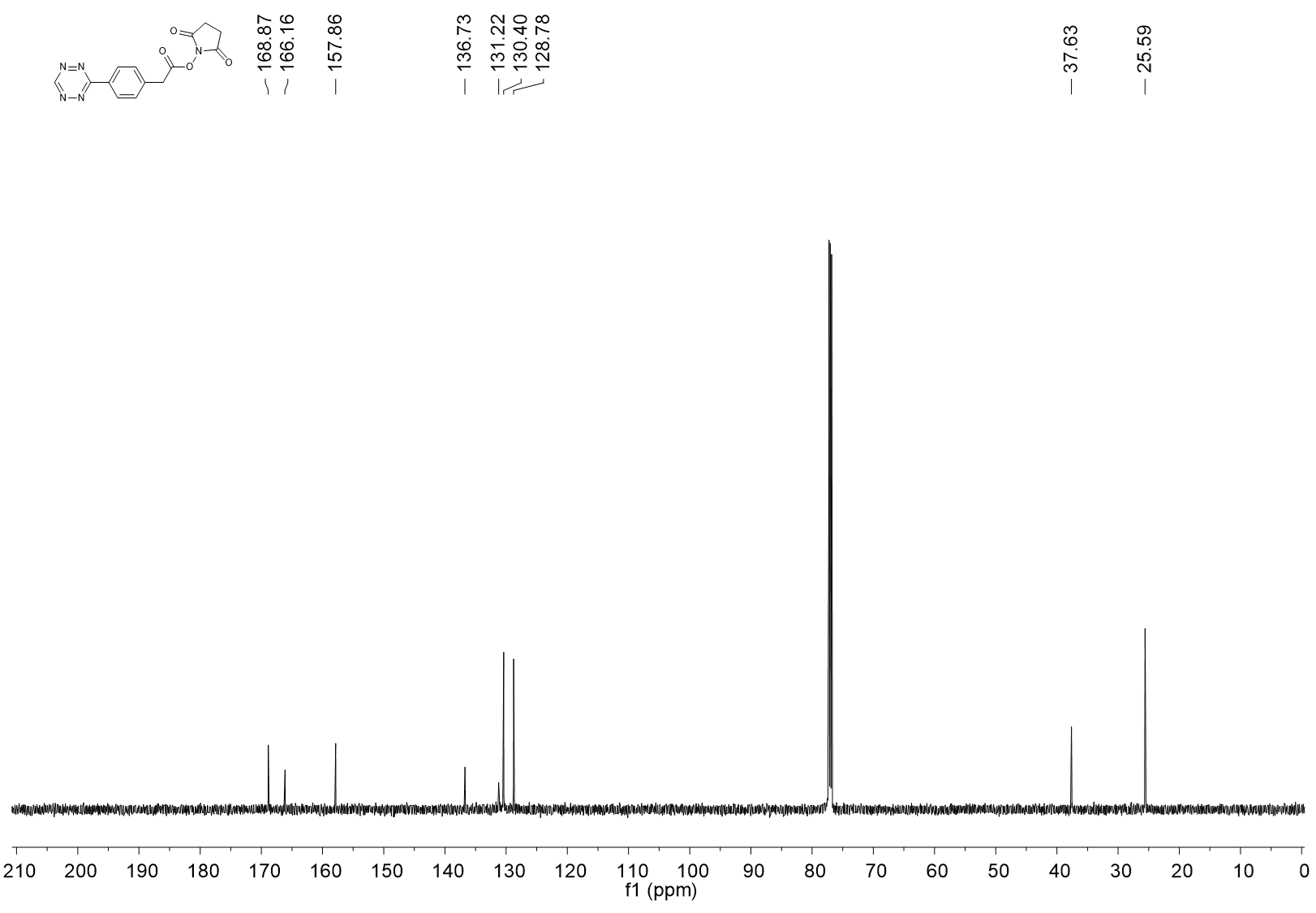


**Figure S4** ^13^C NMR (101 MHz, CDCl_3_) spectrum of **2**.


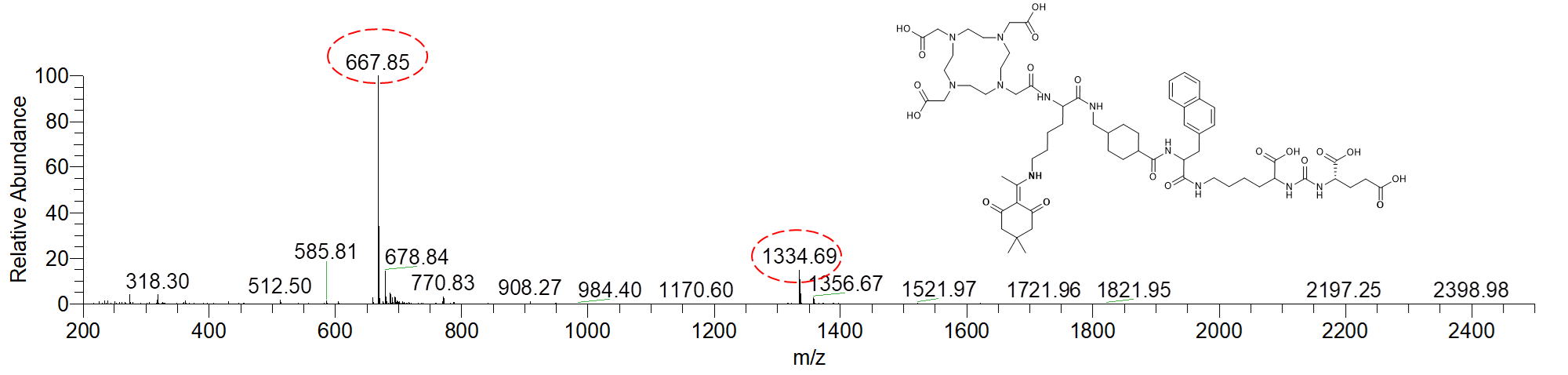


**Figure S5** Mass spectrum of **4**.


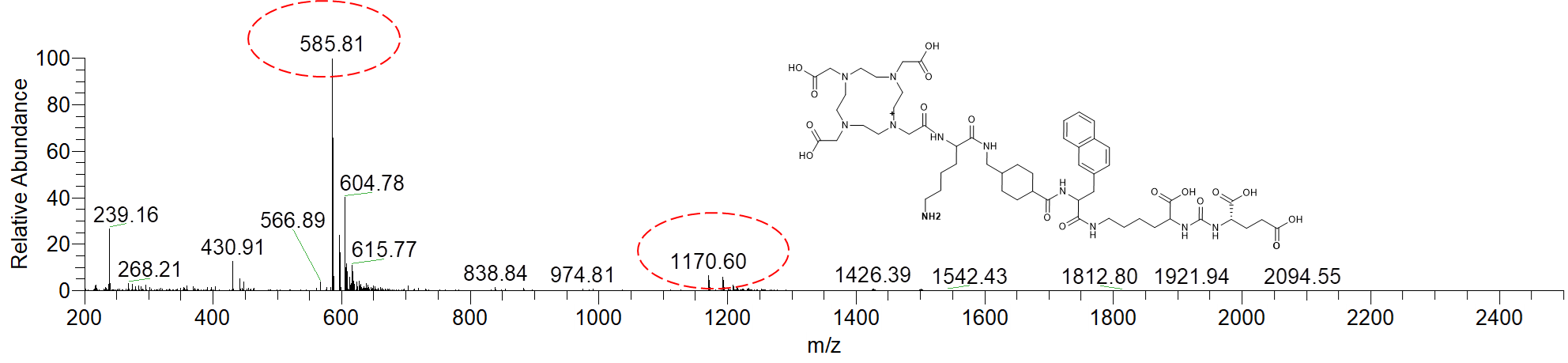


**Figure S6** Mass spectrum of **5**.


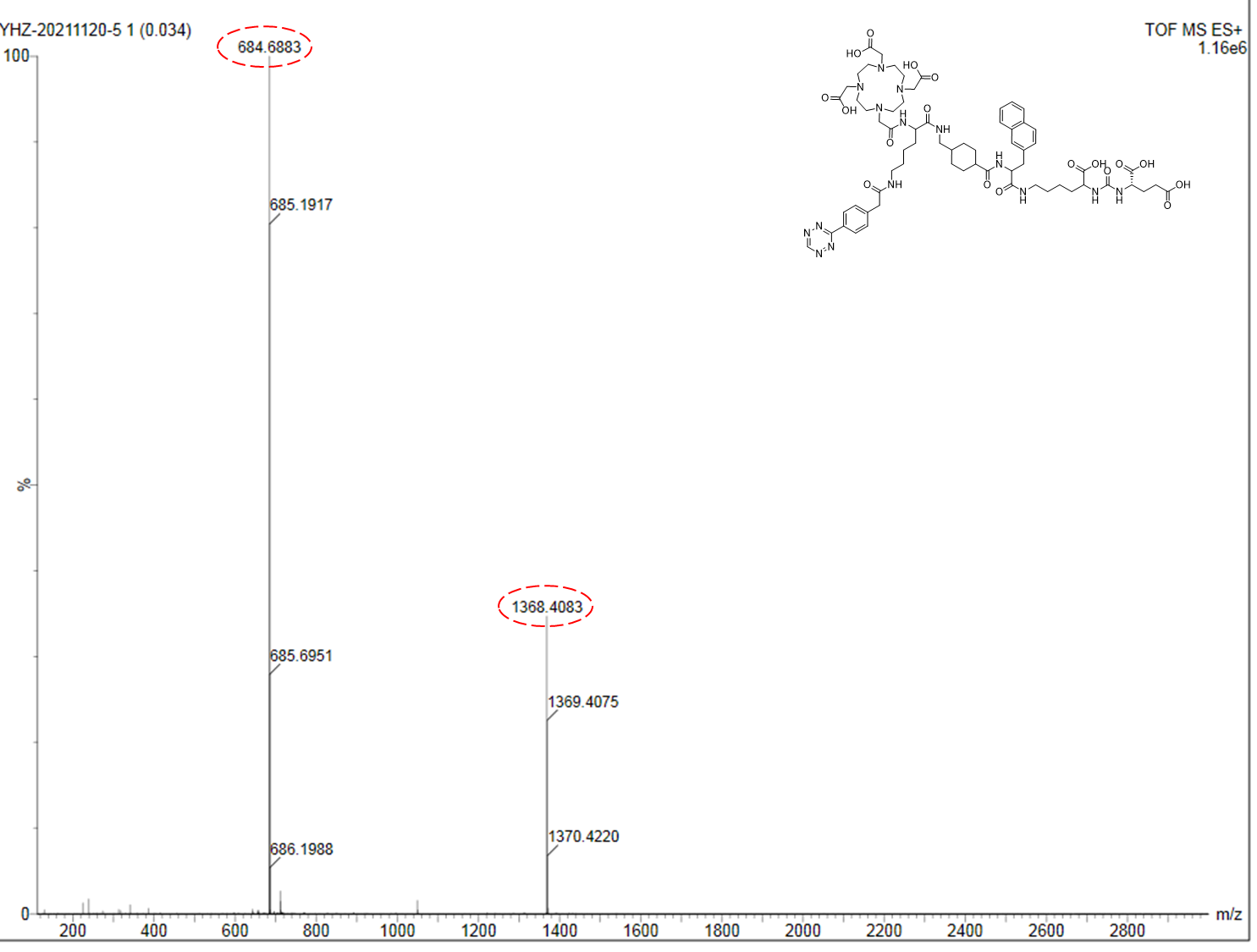


**Figure S7** Mass spectrum of DOTA-PSMA-Tz.


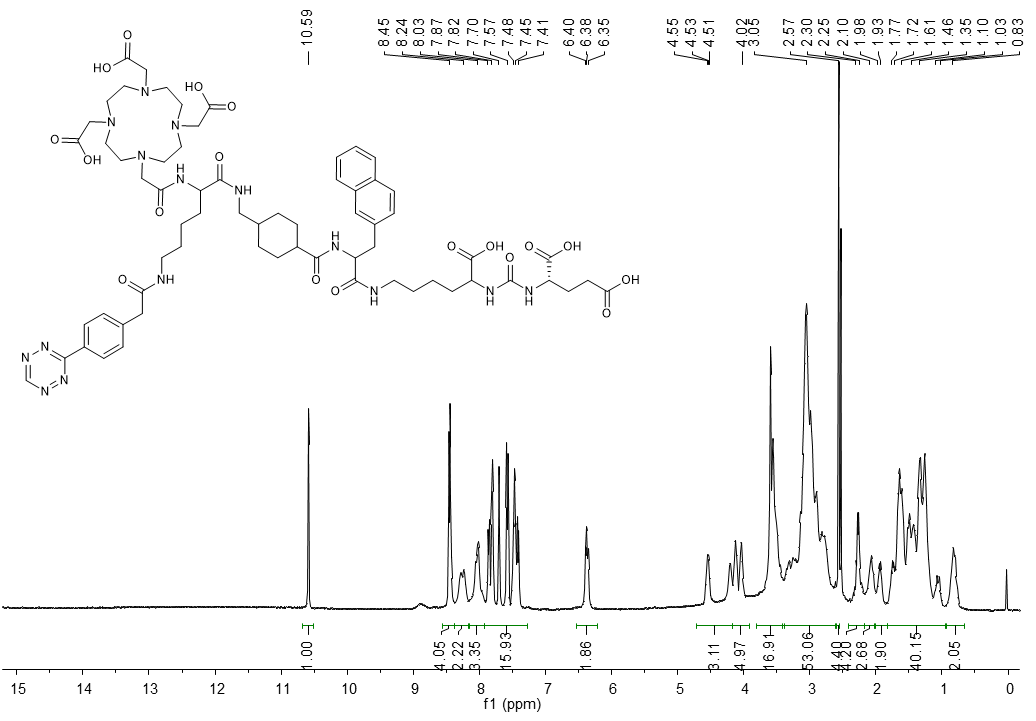


**Figure S8** ^1^H NMR (400 MHz, DMSO) spectrum of DOTA-PSMA-Tz.


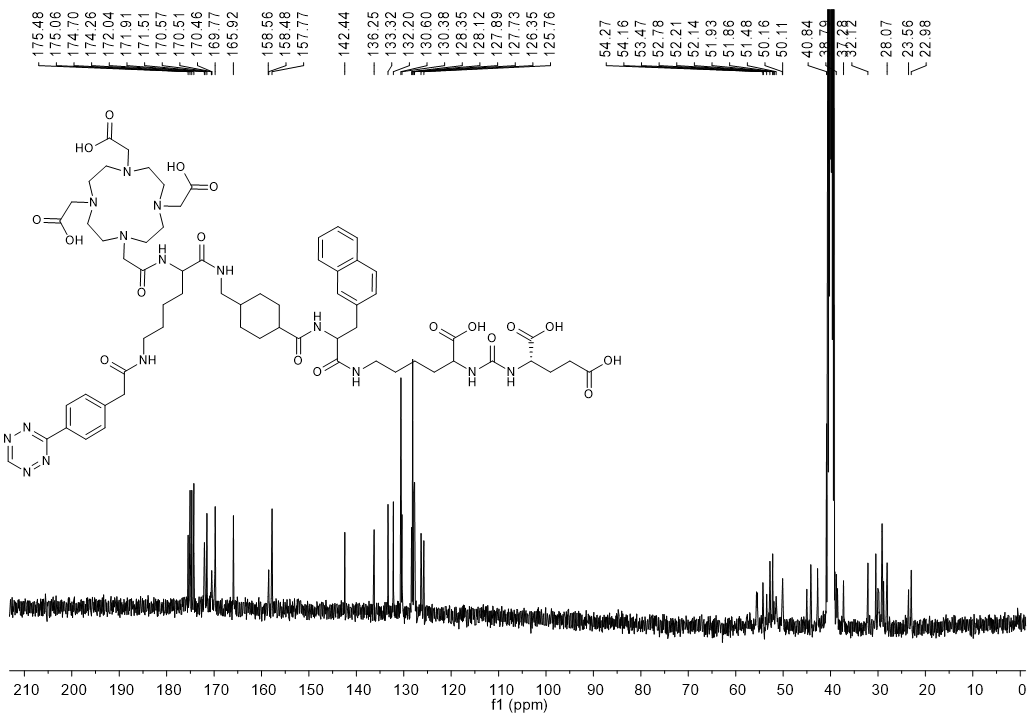


**Figure S9** ^13^C NMR (101 MHz, DMSO) spectrum of DOTA-PSMA-Tz.

1. **Radiochemistry.**

The radiolabeling procedure of [^68^Ga]Ga-PSMA-Tz and [^68^Ga]Ga-PSMA617 was performed as follows: 1 mL of ^68^GaCl_3_ (740 MBq in 0.1 M HCl) was diluted with 0.5 mL of sodium acetate (0.25 M in water, pH = 6.5) to yield ^68^GaCl_3_-sodium acetate solution (pH 4-4.5) and transferred to a vial containing precursors (approximately 0.03 μmol dissolved in 10 μL of dimethyl sulfoxide (DMSO)). The reaction mixture was subsequently heated for 15 min at 95 °C to form [^68^Ga]Ga-PSMA-Tz and [^68^Ga]Ga-PSMA617.

**
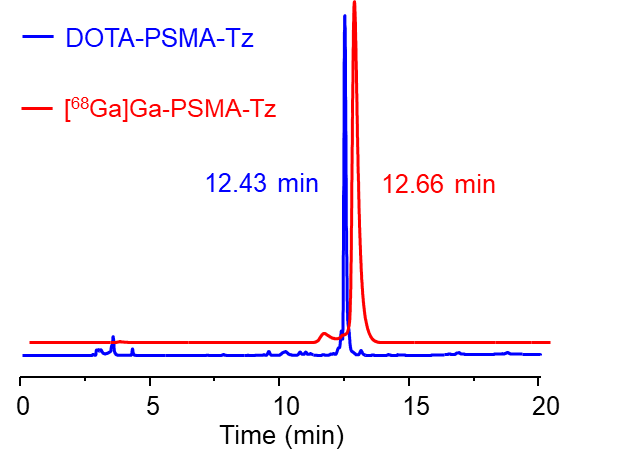
**

**Figure S10** Analytical HPLC chromatogram of [^68^Ga]Ga-PSMA-Tz and DOTA-PSMA-Tz.

The radiolabeling procedure of [^177^Lu]Lu-PSMA-Tz was performed as follows: 740 MBq ^177^LuCl_3_ (in 0.04 M HCl) was diluted with 300 μL of 0.4 M ammonium acetate (pH = 5.6) and transferred into a vial containing 0.03 μmol precursors in 10 μL of DMSO. The vial was subsequently heated for 30 min at 95 °C to form [^177^Lu]Lu-PSMA-Tz. Radiochemical purity was determined using radio-HPLC or iTLC. A C18 reversed-phase column (Hypersil GOLD 250×4.6 mm, 5 μm, Thermo Scientific) was used for HPLC analysis by the following method: a linear gradient eluent starting from 95% A (0.1% TFA in water) and 5% B (0.1% TFA in acetonitrile), increasing to 95% B at a flow rate of 1 mL/min for 25 min, remaining constant at 95% B for another 15 min. The radiochemical purities of corresponding radioligands are >97%.


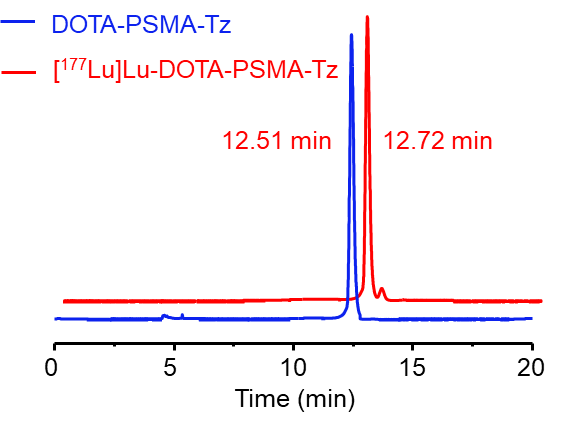


**Figure S11** Analytical HPLC chromatogram of [^177^Lu]Lu-PSMA-Tz and DOTA-PSMA-Tz.

1. **Stability**

**5.1** ***In vitro* stability**

Purified [^68^Ga]Ga-PSMA-Tz (3.7 MBq, 10 μL) was added into 90 μL PBS and serum, respectively. The mixtures were incubated at 37 °C after 0.5, 1, and 2 h, then RCPs were assayed by radio-HPLC. The stabilities in serum were determined in the same way with 90 μL mouse serum added instead. The mixtures were incubated at 37 °C after 0.5, 1, and 2 h. After incubation, 100 μL acetonitrile was added to the mixture, and centrifuged at 16, 543 xg for 8 min. The supernatant was filtrated by a 0.22 μm millipore filter. RCPs of the filtrate were analyzed by radio-HPLC.


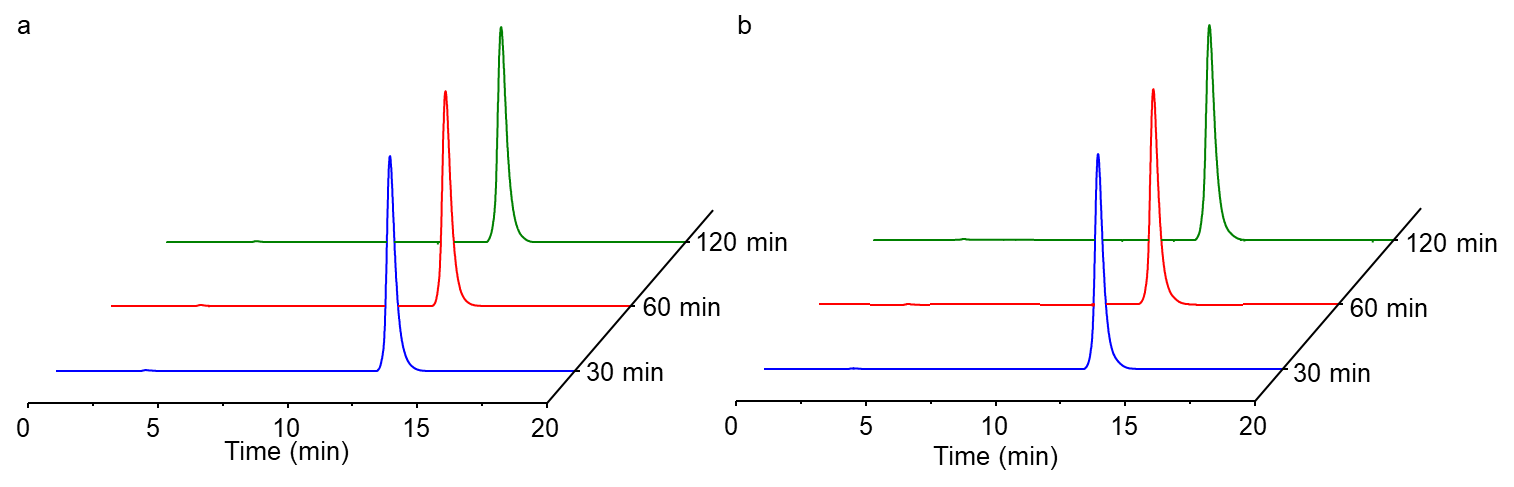


**Figure S12**. Stabilities of [^68^Ga]Ga-PSMA-Tz in PBS (**a**) and serum (**b**).

**5.2** ***In vivo* metabolic stability**

Normal mice were intravenously injected with [^68^Ga]Ga-PSMA-Tz (37 MBq in 200 µL saline) and sacrificed at the indicated time with blood and urine collected. Each blood sample was immediately centrifuged at 10000 rpm for 5 min to obtain the serum. The serum was then treated with acetonitrile and centrifuged at 10000 rpm for 8 min to provide the supernatant. The supernatant was collected and passed through a 0.22 µm Millipore filter. Each urine sample was diluted with 100 µL water and passed through a 0.22 µm millipore filter. All samples were analyzed by radio-HPLC.


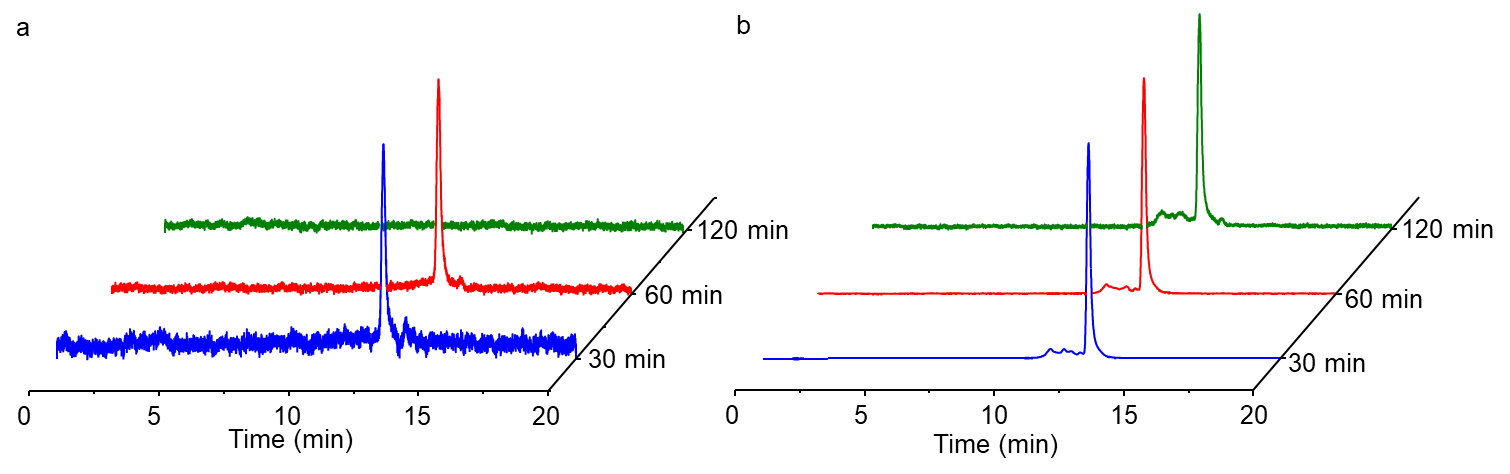


**Figure S13** *In vivo* metabolic stability of [^68^Ga]Ga-PSMA-Tz in healthy C57BL/6 mice through radio-HPLC analysis of blood (a) and urine (b).

1. **Log *D***

The distribution coefficient (log *D*) of [^68^Ga]Ga-PSMA-Tz was measured in a pre-saturated 1-octanol/PBS (pH = 7.4) system. Log *D* was measured following the method: [^68^Ga]Ga-PSMA-Tz (1.11 MBq) dissolved in 100 μL H_2_O and diluted with 0.9 mL PBS (0.05 mol L^-1^, pH = 7.4) and 1 mL 1-octanol. After shaking for 3 min, the mixture was centrifuged at 6000 rpm for 5 min. The counts of the 100 μL organic layer and 100 μL inorganic layer were determined by a gamma counter, respectively. The following equation was used to calculate log *D* = (activity in octanol phase-background activity)/(activity in aqueous phase-background activity). All the experiments were performed with triplicate samples and reported as mean ± SD. The log *D* of [^68^Ga]Ga-PSMA-Tz was determined to be -3,14 ± 0.32.

1. **Saturation binding assays**

**
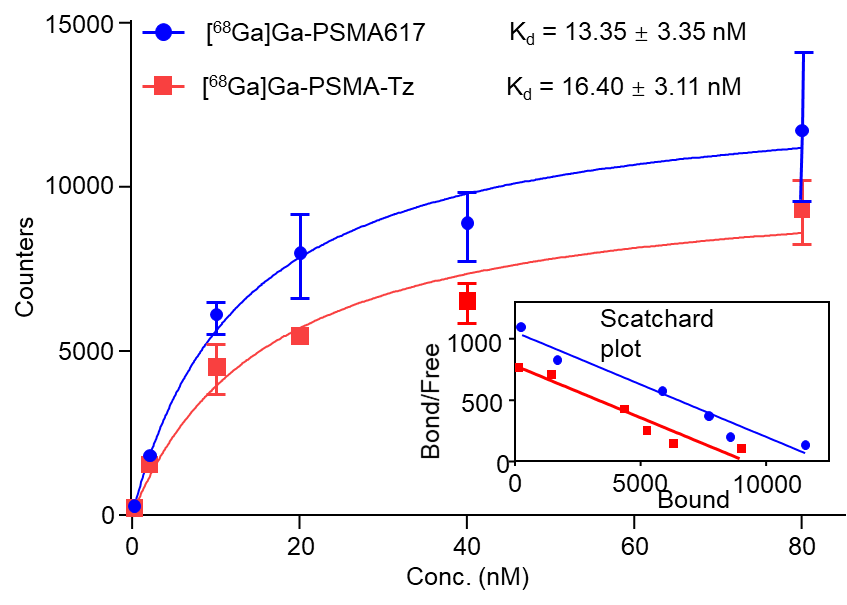
**

**Figure S14.** Saturation binding assay of [^68^Ga]Ga-PSMA617 and [^68^Ga]Ga-PSMA-Tz. All data are shown as mean ± SD (*n* = 4).

1. **Time-dependent cell uptake**

**
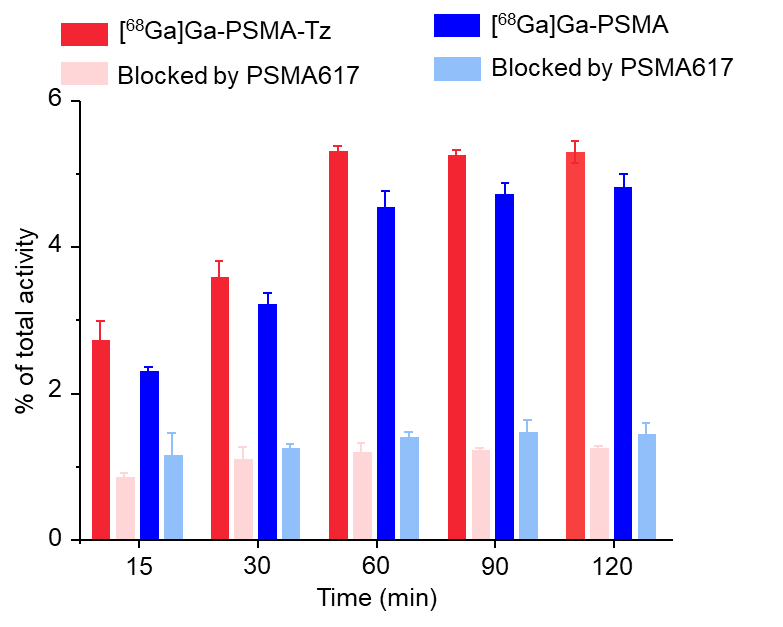
**

**Figure S15.** The specific uptake of [^68^Ga]Ga-PSMA-Tz and [^68^Ga]Ga-PSMA617. All data are shown as mean ± SD (*n* = 4).

1. ***In vitro* stability of Pd@Au-PEG-TCO**

The UV-Vis-NIR absorption spectra of Pd@Au-PEG-TCO were recorded using the Cary 5000 Scan UV-Vis-NIR spectrophotometer (Varian Medical Systems, Palo Alto, CA, USA) at day 0, 1, 3, 5, and 7, respectively.


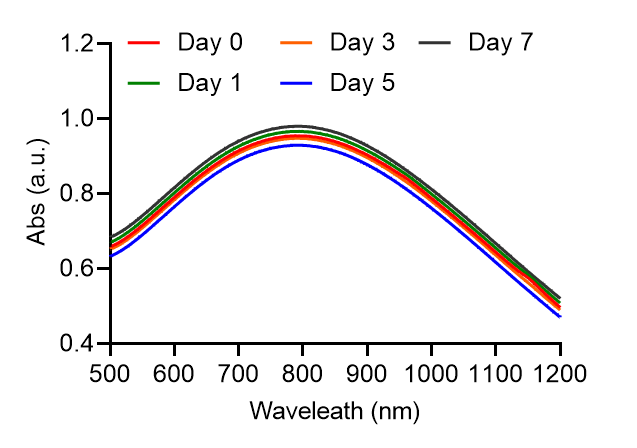


**Figure S16** The absorption spectrum of Pd@Au-PEG-TCO after incubation in the saline for 0, 1, 3, 5, and 7 d, respectively.

1. **Biodistribution of Pd@Au-PEG-TCO**

**Figure S17.** Biodistribution of Pd@Au-PEG in different organs at different times. All data are shown as mean ± SD (*n* = 4)

1. **PA imaging**

Photoacoustic imaging was performed using Visualsonics LAZR-X Vevo (Fujifilm, Japan). Pd@Au-PEG-TCO (200 μg/200 μL) was injected into 22Rv1 xenograft models (*n* = 3) *via* the tail vein. The cross-section signals of the tumor were monitored using a VisualSonics LAZR-X Vevo detector at 0, 2, 4, 8, 12, and 24 h p.i.
